# miR-1915 and miR-1225-5p Regulate the Expression of CD133, PAX2 and TLR2 in Adult Renal Progenitor Cells

**DOI:** 10.1371/journal.pone.0068296

**Published:** 2013-07-08

**Authors:** Fabio Sallustio, Grazia Serino, Vincenzo Costantino, Claudia Curci, Sharon N. Cox, Giuseppe De Palma, Francesco P. Schena

**Affiliations:** 1 Department of Emergency and Organ Transplantation, University of Bari, Bari, Italy; 2 C.A.R.S.O. Consortium, Valenzano, Bari, Italy; 3 Department of Science, Biological and Environmental Sciences and Technologies, University of Salento, Lecce, Italy; 4 Schena Foundation, European Research Center for Kidney Diseases, Valenzano, Bari, Italy; University of Torino, Italy

## Abstract

Adult renal progenitor cells (ARPCs) were recently identified in the cortex of the renal parenchyma and it was demonstrated that they were positive for PAX2, CD133, CD24 and exhibited multipotent differentiation ability. Recent studies on stem cells indicated that microRNAs (miRNAs), a class of noncoding small RNAs that participate in the regulation of gene expression, may play a key role in stem cell self-renewal and differentiation. Distinct sets of miRNAs are specifically expressed in pluripotent stem cells but not in adult tissues, suggesting a role for miRNAs in stem cell self-renewal. We compared miRNA expression profiles of ARPCs with that of mesenchymal stem cells (MSCs) and renal proximal tubular cells (RPTECs) finding distinct sets of miRNAs that were specifically expressed in ARPCs. In particular, miR-1915 and miR-1225-5p regulated the expression of important markers of renal progenitors, such as CD133 and PAX2, and important genes involved in the repair mechanisms of ARPCs, such as TLR2. We demonstrated that the expression of both the renal stem cell markers CD133 and PAX2 depends on lower miR-1915 levels and that the increase of miR-1915 levels improved capacity of ARPCs to differentiate into adipocyte-like and epithelial-like cells. Finally, we found that the low levels of miR-1225-5p were responsible for high TLR2 expression in ARPCs. Therefore, together, miR-1915 and miR-1225-5p seem to regulate important traits of renal progenitors: the stemness and the repair capacity.

## Introduction

In the last years regenerative medicine was mainly directed towards the use of adult stem cells to improve the repair of injured organs. This trend was observed within various medical segments, including nephrology [1]–[3]. In particular many researchers focused their attention on the possibility of using adult renal stem/progenitor cells (ARPCs) for regenerative purposes. These cells exhibited multipotent differentiation ability by generating tubular epithelial-like, osteogenic-like, adipocyte-like, and neuronal-like cells. When injected into mice with glycerol-induced acute renal injury, these CD133+/CD24+ ARPCs contributed to tubular regeneration [1], [4]–[6].

ARPCs, first identified in the renal interstitium and then in Bowman’s capsule, are positive for PAX2, CD133 and CD24 [4], [5], [6]–[8]. Their expression profiles and their phenotypical characteristics are very similar [7] but some specific properties as the inclination of tubular cells to proliferate upon tubular injury in patients with acute or chronic tubular damage were different [6], [7], [9]. Moreover, markers allowing distinction between glomerular and tubular progenitor subpopulations have been recently identified [6].

Recent studies have indicated that microRNAs (miRNAs), a class of noncoding small RNAs that participate in the regulation of gene expression, may play a key role in stem cell self-renewal and differentiation [10]. MicroRNAs are specifically attractive candidates for regulating stem cell identity, which includes self-renewal and cell fate decisions, as their ability to simultaneously regulate many targets provides a means for coordinated control of gene action. Although direct evidence for a functional role for miRNAs in stem cell biology is just emerging, exciting hints regarding their involvement based on expression patterns, predicted targets, and over-expression studies suggest that miRNAs may be one of the key regulators [11]. Distinct sets of miRNAs are specifically expressed in pluripotent stem cells but not in adult tissues, suggesting a role for miRNAs in stem cell self-renewal [12], [13].

Expression level of several miRNAs has been correlated to the time of stem cell differentiation, suggesting that these miRNAs could be used as markers to monitor stem cell identity and differentiation [12]–[14]. Furthermore, discovery of both stem cell differentiation-related miRNAs and their potential target mRNA genes may provide further insights about their functional roles in stem cell maintenance and differentiation. To date, relatively little is known about functions of miRNAs in the kidney and in particular in ARPCs.

In this study we compared miRNA expression profiles of renal progenitors with that of mesenchymal stem cells (MSCs) and of renal proximal tubular epithelial cells (RPTECs) and found distinct sets of miRNAs that were specifically expressed in ARPCs. In particular, miR-1915 and miR-1225-5p regulated the expression of important markers of renal progenitors, such as CD133 and PAX2, and important genes involved in the repair mechanisms of ARPCs, such as TLR2.

## Results

### Isolation and Characterization of ARPCs

**Figure 1:**
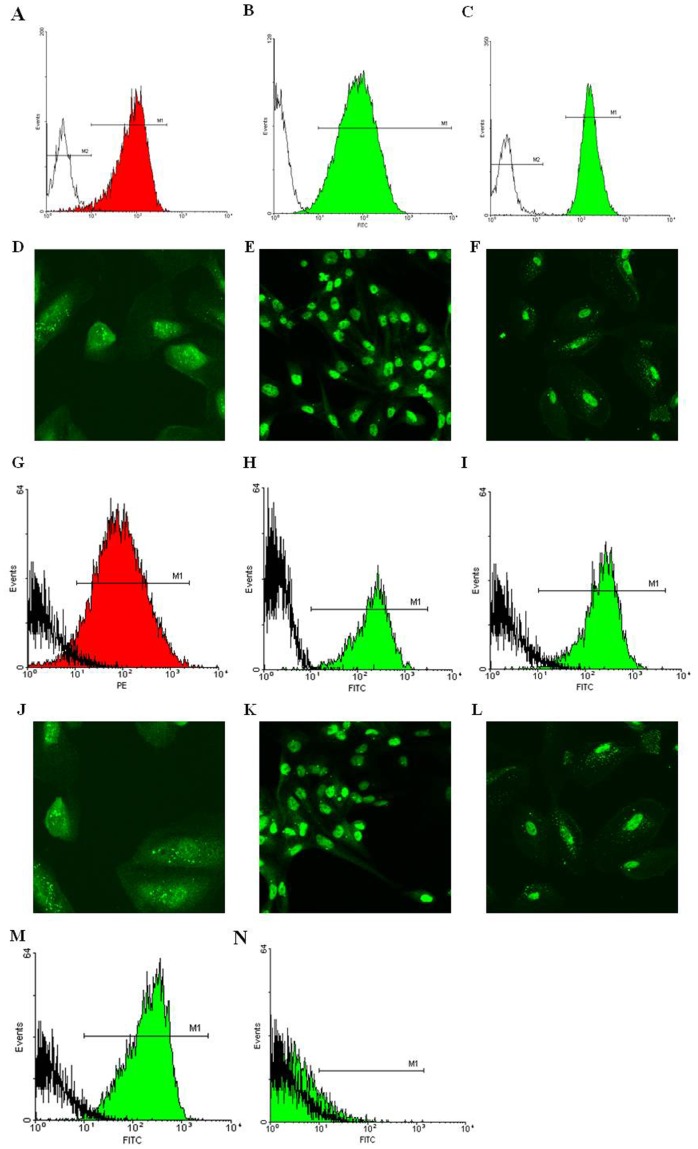
Characterization of isolated glomerular and tubular ARPCs. Cytofluorimetric and immunofluorescence analysis of tARPCs showed the expression of: CD133 (**A**), CD24 (**B**), CD44 (**C**), Oct-4 (**D**), PAX2 (**E**), BMI-1 (**F**), Cytofluorimetric and immunofluorescence analysis of gARPCs showed the expression of: CD133 (**G**), CD24 (**H**), CD44 (**I**), Oct-4 (**J**), PAX2 (**K**), BMI-1 (**L**). CD106 expression in glomerular (**M**) and tubular (**N**) ARPCs. Original view: X63.

CD133-positive ARPCs were isolated, by magnetic sorting, from glomerular and tubular fractions of healthy cortex of kidney removed for renal carcinoma. Both ARPCs isolated from glomeruli (gARPCs) and from tubular compartment (tARPCs) were positive for CD133, CD24, PAX2, BMI-1, Oct-4 and CD44 (Figure 1A–F, J–L), previously described as markers of adult renal progenitors [3]–[5], [6], [15]. However, gARPCs were positive for the CD106 (Vascular Cell Adhesion Molecule 1, VCAM1) expression (Figure 1M), whereas tARPCs did not express the CD106 marker (Figure 1N). However, CD34, CD105, and CD45 membrane proteins were not detectable (data not shown).

### Identification of Differentially Expressed miRNAs in tARPCs, gARPCs and MSCs Compared to RPTECs

**Figure 2:**
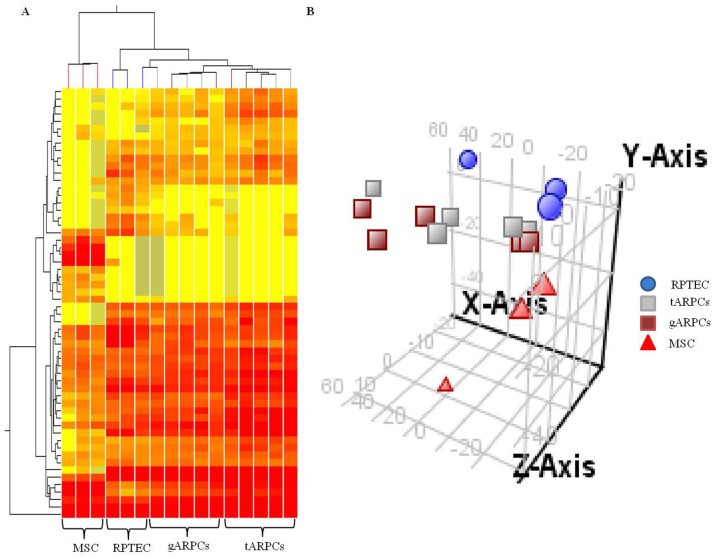
Unsupervised hierarchical clustering and principal component analysis (PCA) of miRNA expression profile. miRNA expression patterns of 5 tARPC, 5 gARPC and 3 MSC different clones and 3 different RPTEC lines were examined using Agilent array composed of 1205 human miRNAs. A total of 327 miRNA resulted expressed between different cell types (false discovery rate <0.01). The 2-D hierarchical clustering (**A**) and the PCA (**B**) showed that miRNA expression profile was different among MSCs, RPTECs and ARPCs, whereas it was very similar between gARPCs and tARPCs.

To identify miRNAs typically expressed in ARPCs, we analyzed their global expression profile in 5 different tARPC and gARPC clones by microarray technology and compared them with RPTEC and MSC global miRNA expression. Among 1205 human miRNAs represented on the microarrays, we found 327 miRNAs that resulted expressed between different cell types. The 2-D hierarchical clustering and the principal component analysis showed that miRNA expression profile was different among MSCs, RPTECs and ARPCs, whereas it was very similar between gARPCs and tARPCs (Figure 2A and 2B), even if statistical analysis identified some little differences. In fact, applying the Benjamini-Hochberg statistical method and selecting genes modulated with a fold change of 2, we identified 21 miRNAs discriminating tARPCs from RPTECs, 41 miRNAs discriminating gARPCs from RPTECs, 24 miRNAs discriminating tARPCs from MSCs, 31 miRNAs discriminating gARPCs from MSCs and 30 miRNAs discriminating MSCs from RPTECs (Table 1). When we compared miRNAs modulated in tARPCs and gARPCs in respect to RPTECs with a fold change of 2, we found 14 miRNAs that were jointly modulated. However, 7 miRNAs were specifically modulated in tARPCs and 27 in gARPCs (Table 2). Moreover, comparing miRNA expression of MSCs with that of tARPCs and gARPCs, we found 5 miRNAs varying in ARPCs in comparison to RPTECs that were also modulated in MSCs; 6 miRNAs were in common between gARPCs and MSCs and 19 miRNAs were exclusively modulated in MSCs (Figure S1 in File S1).

**Table 1:** miRNAs modulated between ARPCs, RPTECs and MSCs (FDR<0.01).

tARPCs vs RPTECs		gARPCs vs RPTECs		MSCs vs tARPCs	
miRNA	Fold change	miRNA	Fold change	miRNA	Fold change
hsa-miR-1225-5p	−3,530	hsa-miR-7	9,640	hsa-miR-30a	−7,019
hsa-miR-130b	3,504	hsa-miR-874	−2,033	hsa-miR-196b	−3,328
hsa-miR-93	2,617	hsa-miR-1225-5p	-4,519	hsa-miR-93	−3,155
hsa-miR-16	2,470	hsa-miR-887	−12,854	hsa-miR-92a	−5,353
hsa-miR-193b	2,299	hsa-miR-625	4,770	hsa-miR-19b	−14,477
hsa-miR-1306	2,777	hsa-miR-130b	3,951	hsa-miR-30b	−7,790
hsa-miR-373*	−4,669	hsa-miR-196b	3,072	hsa-miR-17	−14,038
hsa-miR-1977_v14.0	3,340	hsa-miR-128	2,566	hsa-miR-107	−2,130
hsa-miR-17	3,602	hsa-miR-484	8,361	hsa-miR-30a*	−5,005
hsa-miR-455-3p	2,307	hsa-miR-320c	2,049	hsa-miR-1181	2,554
hsa-miR-1181	−6,379	hsa-miR-16	2,700	hsa-miR-210	−11,421
hsa-miR-1915	−2,185	hsa-miR-92a	2,985	hsa-miR-30c	−7,624
hsa-miR-1226*	−2,616	hsa-miR-193b	2,497	hsa-miR-28-5p	−3,876
hsa-miR-20b	2,916	hsa-miR-320a	2,346	hsa-let-7b	2,360
hsa-miR-371-5p	−3,773	hsa-miR-373*	−19,333	hsa-miR-20b	−6,852
hsa-miR-125b	4,684	hsa-miR-17	3,926	hsa-miR-155	−27,322
hsa-miR-100	5,623	hsa-miR-455-3p	2,954	hsa-miR-125b	14,093
hsa-miR-20a	3,195	hsa-miR-505	4,678	hsa-miR-100	6,149
hsa-miR-15a	2,199	hsa-miR-296-5p	−3,128	hsa-miR-103	−2,469
hsa-miR-25	2,202	hsa-miR-629*	−8,553	hsa-miR-151-3p	−3,364
hsa-miR-15b	2,790	hsa-miR-663	−6,565	hsa-miR-146b-5p	−6,004
		hsa-miR-1181	−24,765	hsa-miR-20a	−8,674
**MSCs vs gARPCs**		hsa-miR-134	−12,748	hsa-miR-30d	−3,394
**miRNA**	**Fold change**	hsa-miR-1915	−2,634	hsa-miR-30e*	−3,492
hsa-miR-1275	−2,0642676	hsa-miR-574-3p	2,791		
hsa-miR-30a	−6,151071	hsa-miR-20b	3,337	**MSCs vs RPTECs**	
hsa-miR-31	−3,471138	hsa-miR-371-5p	−5,501	**miRNA**	**Fold change**
hsa-miR-196b	−4,8021374	hsa-miR-1207-5p	−3,784	hsa-miR-874	−2,7487714
hsa-miR-331-3p	−2,5886998	hsa-miR-718	−12,757	hsa-miR-1225-5p	−7,3637238
hsa-miR-193a-5p	3,0352206	hsa-miR-1469	−3,469	hsa-let-7i	4,453724
hsa-miR-342-3p	−2,4045403	hsa-miR-125b	7,029	hsa-miR-30a	−7,8995056
hsa-miR-92a	−6,043755	hsa-miR-196a	2,285	hsa-miR-193a-5p	3,3988273
hsa-miR-30b	−7,9313436	hsa-miR-100	8,157	hsa-miR-320c	2,208497
hsa-miR-17	−15,299799	hsa-miR-197	2,294	hsa-miR-193b	2,5278797
hsa-miR-455-3p	−2,5227208	hsa-miR-150*	−5,103	hsa-miR-30b	−6,955626
hsa-miR-107	−2,339731	hsa-miR-181a*	5,393	hsa-miR-30a*	−4,9433923
hsa-miR-30a*	−5,1749806	hsa-miR-320b	2,310	hsa-miR-99a	3,9254699
hsa-miR-210	−11,182305	hsa-miR-151-3p	2,060	hsa-miR-29b-1*	2,093193
hsa-miR-425	−2,2963724	hsa-miR-320d	2,029	hsa-miR-30c	−9,263635
hsa-miR-361-5p	−2,0869288	hsa-miR-602	−3,607	hsa-miR-28-5p	−2,423316
hsa-miR-30c	−8,070546	hsa-miR-15b	3,372	hsa-let-7b	2,5691411
hsa-miR-28-5p	−4,199433			hsa-miR-140-5p	3,055944
hsa-let-7b	2,8582647			hsa-miR-20b	−2,3495286
hsa-miR-20b	−7,840423			hsa-miR-29a	2,2842536
hsa-miR-155	−29,704147			hsa-miR-1207-5p	−5,718977
hsa-let-7c	2,1411626			hsa-miR-155	−24,928856
hsa-miR-125b	9,391929			hsa-miR-23a	2,029089
hsa-miR-196a	−2,167844			hsa-miR-221*	3,449269
hsa-miR-103	−2,7692318			hsa-miR-125b	66,01536
hsa-miR-150*	−2,4807231			hsa-miR-100	34,57493
hsa-miR-151-3p	−3,4726465			hsa-miR-10a	−3,0170193
hsa-miR-146b-5p	−4,659217			hsa-miR-150*	−12,65959
hsa-miR-20a	−10,063658			hsa-miR-320b	2,068215
hsa-miR-30d	−3,3238764			hsa-miR-140-3p	2,9847937
hsa-miR-30e*	−4,100744			hsa-miR-30d	−2,8532631
				hsa-miR-320d	2,2541873
				hsa-miR-30e*	−3,731827

**Table 2:** miRNAs specifically modulated in tARPCs and gARPCs.

Common miRNAs tARPCs gARPCs vs RPTECs		gARPC specific miRNAs vs RPTECs	
miRNA	Fold change	miRNA	Fold change
hsa-miR-1225-5p	−3,530	hsa-miR-7	9,640
hsa-miR-130b	3,504	hsa-miR-874	−2,033
hsa-miR-16	2,470	hsa-miR-887	−12,854
hsa-miR-193b	2,299	hsa-miR-625	4,770
hsa-miR-373*	−4,669	hsa-miR-196b	3,072
hsa-miR-17	3,602	hsa-miR-128	2,566
hsa-miR-455-3p	2,307	hsa-miR-484	8,361
hsa-miR-1181	−6,379	hsa-miR-320c	2,049
hsa-miR-1915	−2,185	hsa-miR-92a	2,985
hsa-miR-20b	2,916	hsa-miR-320a	2,346
hsa-miR-371-5p	−3,773	hsa-miR-505	4,678
hsa-miR-125b	4,684	hsa-miR-296-5p	−3,128
hsa-miR-100	5,623	hsa-miR-629*	−8,553
hsa-miR-15b	2,790	hsa-miR-663	−6,565
		hsa-miR-134	−12,748
**tARPC specific miRNAs vs RPTECs**		hsa-miR-574-3p	2,791
**miRNA**	**Fold change**	hsa-miR-1207-5p	−3,784
hsa-miR-93	2,617	hsa-miR-718	−12,757
hsa-miR-1306	2,777	hsa-miR-1469	−3,469
hsa-miR-1977_v14.0	3,340	hsa-miR-196a	2,285
hsa-miR-1226*	−2,616	hsa-miR-197	2,294
hsa-miR-20a	3,195	hsa-miR-150*	−5,103
hsa-miR-15a	2,199	hsa-miR-181a*	5,393
hsa-miR-25	2,202	hsa-miR-320b	2,310
		hsa-miR-151-3p	2,060
		hsa-miR-320d	2,029
		hsa-miR-602	−3,607

### Identification of miRNA Targets

**Figure 3:**
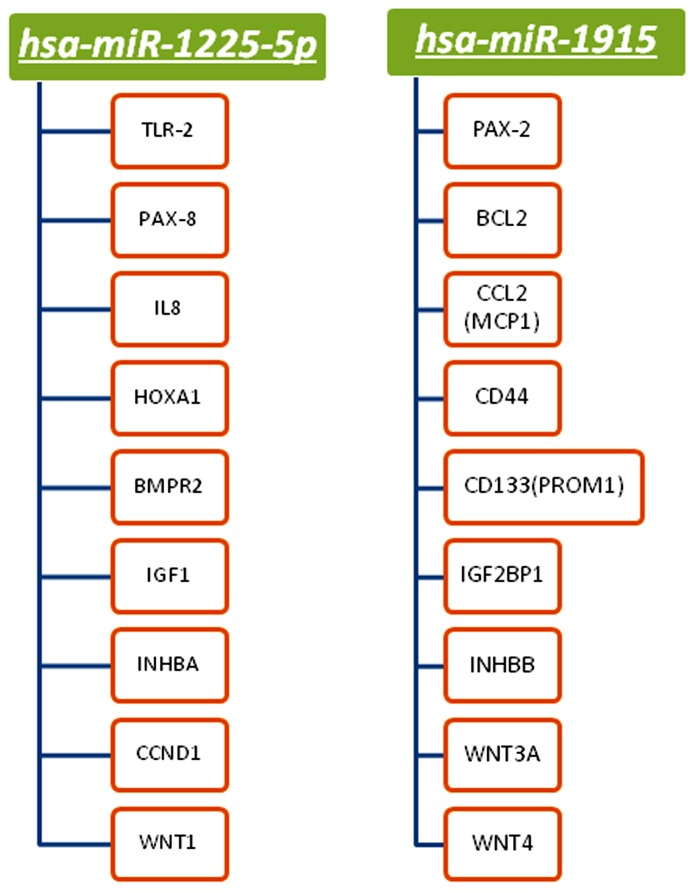
Principal genes predicted to be targeted by miR-1225-5p and miR-1915.

To study the molecular mechanisms in which miRNAs are involved, we performed a bioinformatic analysis to predict target genes of the modulated miRNAs. To reduce the number of false positives, we used 4 different algorithms and potential targets were selected overlapping results and selecting gene targets predicted by at least 2 of them. Based on the results of bioinformatics analysis we found several genes, of great interest for the stem cell biology, that were potentially targeted by some of modulated miRNAs. In particular, among miRNAs modulated respect to RPTECs, we found miR-1225-5p and miR-1915 that were down-regulated in tARPCs and gARPCs and predicted to regulate genes involved in the stemness property of ARPCs, as CD133 and renal embryonic transcription factors PAX2 and PAX8, that are markers of adult renal progenitors, the hyaluronic acid receptor CD44, the toll-like receptor 2 (TLR2), IL-8, HOXA1, some WNT genes and other chemokines and growth factors (Figure 3, Table S1). All these genes have been previously found upregulated in ARPCs and have an important role in specific biological function and stemness characteristics of ARPCs [1], [4]–[5], [7]–[9], [16].

### Validation of miRNA Expression and in silico Analysis of miRNA Targets

**Figure 4:**
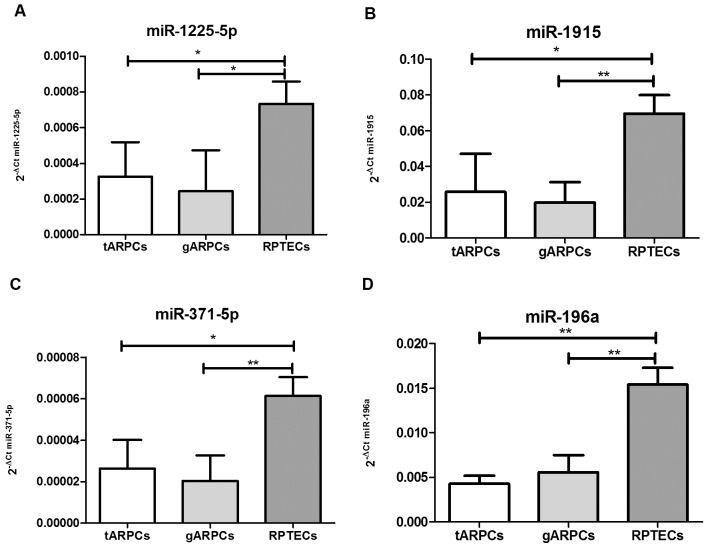
Validation of miRNA expression levels by qRT-PCR. The amount of miR-1225-5p, miR-1915, miR-371-5p and miR-196a in an independent set of 5 tARPC, 5 gARPC clones and 3 RPTEC lines was evaluated by real-time PCR (q-RT-PCR). The miRNA relative expressions were normalized to the expression of U6. Expression levels of miR-1225-5p, miR-1915, miR-371-5p and miR-196a were found significantly lower in tARPCs and gARPCs compared to RPTECs. The histograms represent the mean ± SD. *p<0.03; **p<0.01.

To validate expression results of miRNAs whose putative target genes were more connected with stem cell biology, we performed real-time PCR (q-RT-PCR) for *miR-1225-5p, miR-1915, miR-371-5p and miR-196a* on miRNAs isolated from ARPCs of an independent set of 5 tARPCs, 5 gARPCs clones and 3 RPTEC lines. The expression of all analyzed miRNAs was significantly lower in stem/progenitor cell lines, confirming microarray results (Figure 4).

To deepen the biological interaction among miRNAs and among genes targeted by miRNAs that were found modulated in ARPCs, we performed a new bioinformatics analysis on pathways and biological processes in which target genes of miRNAs were involved. We considered target genes of miRNAs that were jointly modulated in tARPCs and gARPCs respect to RPTECs and genes-miRNA pairs with inverse expression relationships. Among significant canonical pathways we found WNT/β-catenin signalling, ERK5 signalling, growth hormone signalling and glutamate metabolism (Figure S2A in File S1), however among significant biological processes we found cellular growth proliferation, cellular and organismal development, cell morphology and cell-to-cell signalling and interaction (Figure S2B in File S1). Moreover, merging miRNAs with ARPC modulated genes resulting from the gene expression profile published in our previous work [7] (GEO Series Accession Number GSE8611), we found that miRNAs were strongly interconnected with the expressed mRNAs with an inverse pairs expression relationships (Table S2) and two specific significant networks, involving the WNT/B-catenin and Frizzled signalling, were identified (Figure S3 and S4 in File S1). Then, we studied the biological interaction among miRNAs modulated in MSCs and genes targeted by miRNAs, again taking advantage of our previous gene expression data. We considered only genes-miRNA pairs with inverse expression relationships. Among significant canonical pathways we found Inhibition of matrix metalloproteases, WNT/β-catenin signalling, PTEN signalling, regulation of the epithelial-mesenchymal transition (Figure S5A in File S1). Among significant biological processes we found cellular movement, cellular growth proliferation, cell death and survival, cellular and organismal development, cell morphology and cell-to-cell signalling and interaction (Figure S5B in File S1). miRNAs and genes more contributing to these biological processes formed two significant networks represented in Figure S6 and S7 in File S1.

### miR-1915 Regulates both the CD133 and PAX2 Expression in tARPCs

**Figure 5:**
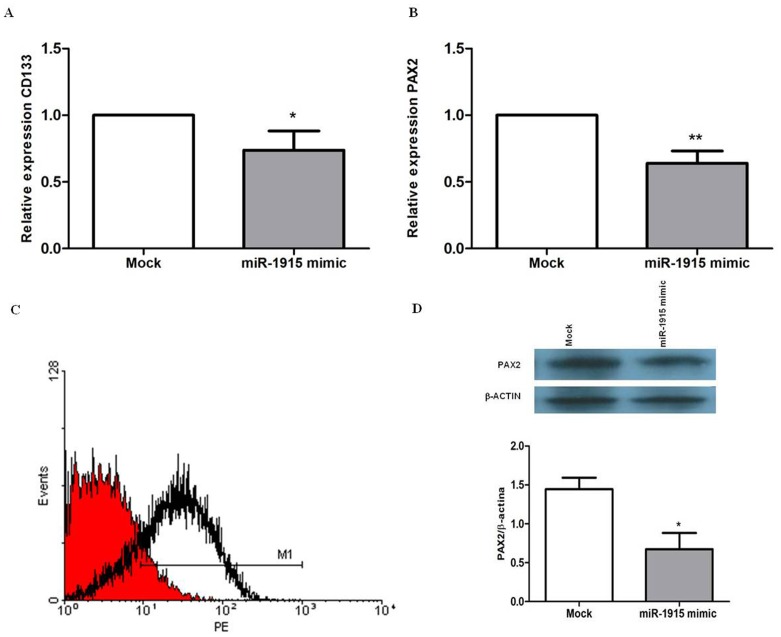
miR-1915 regulates CD133 and PAX2 in ARPCs. (**A–B**) CD133 and PAX2 expression levels were analyzed by real-time PCR following transfection with miR-1915 mimic. Increasing the amount of miR-1915 within ARPCs resulted in a 1.5 fold reduction of both CD133 and PAX2 mRNA levels. Expression data were normalized on the housekeeping gene β-actin. Data are representative of four independent experiments (means ± SEM), *p<0.01; **p<0.0003. (**C**) Surface marker expression of CD133, as measured by flow cytometry, resulted in a large reduction (8% vs 96% of mock transfection control) following transfection with 50 nM miR-1915 mimic. Red area represents the transfected condition. Data are representative of three independent experiments (D) Transfection of ARPCs with 100 nM miR-1915 mimic resulted in a 2 fold reduction of PAX2 protein expression, as shown by Western blot. β-actin was used as endogenous control. Data are representative of three independent experiments (means ± SEM). *p<0.02. Mock indicates mock-transfected cells going through the transfection processes without addition of mimic miRNA.

The CD133 is known as the most important marker of renal progenitor cells, however the genetic basis of its expression in stem cells are unknown. Our *in silico* analysis showed that the miR-1915 downregulation in ARPCs in respect to differentiated renal tubular cells could be the cause of the CD133 overexpression. To confirm that the miR-1915 was able to modulate the expression of the CD133 mRNA, we performed transient transfection experiments using an independent group tARPC clones. We increased the amount of the endogenous miR-1915 within cells, transfecting short RNA sequences that mimic the action of the miRNA, in order to simulate the situation found in RPTECs, that have higher levels of miR-1915. tARPCs, transfected with 25 nM miR-1915 mimic, showed a 1.5 fold reduction of endogenous CD133 mRNA levels (p<0.01, Figure 5A). Since the same miRNA was found by bioinfomatic analysis to target also PAX2 mRNA, we checked whether miR-1915 mimic lowered also its levels and found that transfection with 50 nM miR-1915 mimic caused a 1.5 fold PAX2 mRNA levels reduction too (p<0.0003, Figure 5B). Moreover, transfecting tARPCs with the 50 nM miR-1915 mimic twice at a distance of three days, on the sixth day from the first transfection we obtained a large reduction of CD133 protein surface expression (8% vs 96% of mock transfection control, Figure 5C). The transiently transfection of renal progenitors with 100 nM miR-1915 mimic led also to strong decrease of endogenous PAX2 protein expression (2 fold, p<0.02 Figure 5D) on the sixth day following miRNA mimic induction. All together our results showed that miR-1915 regulated the expression of both CD133 and PAX2, the two most important stemness markers of ARPCs.

### miR-1915 Upregulation Favors tARPC Differentiation

**Figure 6:**
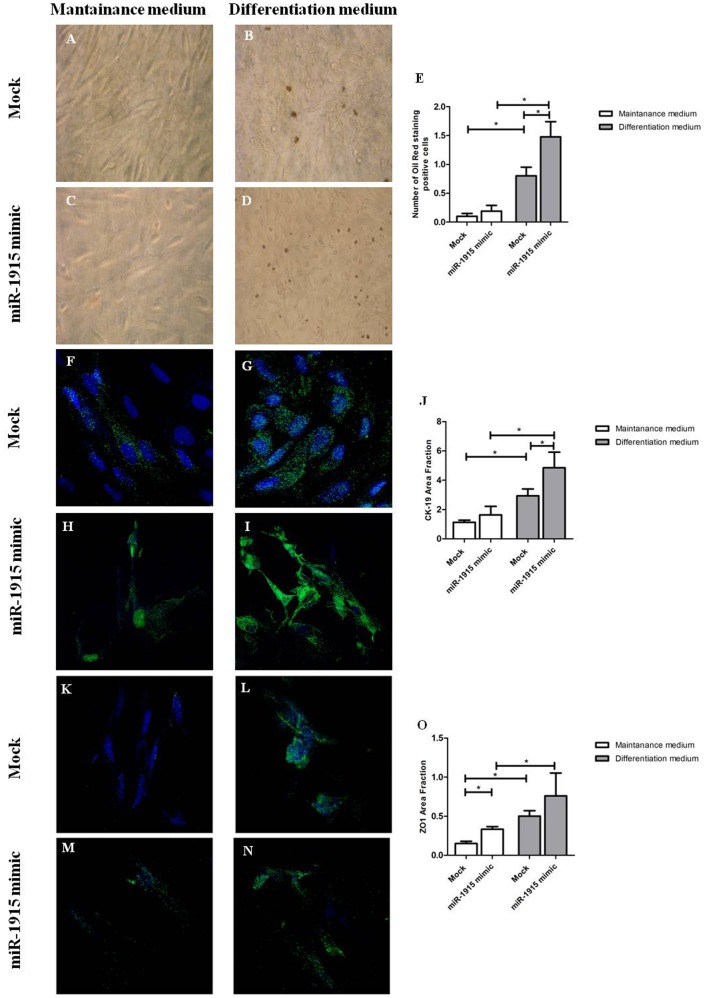
miR-1915 upregulation favors ARPC differentiation. (**A–D**) Effect of miR-1915 on adipocyte differentiation of ARPCs. Cells were cultured for 20 days in adipogenic medium with or without miR-1915 mimic (50 nM). Oil red O staining was used to detect mature adipocytes. The increase of miR-1915 levels resulted in an higher number of adipocyte-like cells (**B, D, E**). (**A, C**) Representative micrographs showing ARPCs cultured in maintenance medium transfected and not-transfected with miR-1915 mimic. (**E**) The number of adipocyte was counted and reported as a percentage of total ARPCs. Values are expressed as means ± SEM. *p<0.01. (**F–J**) ARPC differentiation in epithelial-like cells with and without miR-1915 (50 nM) and stained positively for CK-19. The increase of miR-1915 levels resulted in an higher CK-19 expression (**G, I, J**). (**F, H**) Representative micrographs showing ARPCs cultured in maintenance medium transfected and not-transfected with miR-1915 mimic. (**K–O**) ARPC differentiation in epithelial-like cells with and without miR-1915 (50 nM) and stained positively for ZO-1. (**K, M**) Representative micrographs showing ARPCs cultured in maintenance medium transfected and not-transfected with miR-1915 mimic. To-pro-3 counterstains nuclei (blue). Original view X63. *p<0.01 vs. not differentiated cells. Mock indicates mock-transfected cells going through the transfection processes without addition of mimic miRNA.

To study whether miR-1915, affecting CD133 and PAX2 expression, influenced also renal progenitor differentiation capacity, we increased miR-1915 levels in tARPCs by mimic transfection (50 nM miR-1915 mimic) and induced their differentiation towards epithelial or adipocytic phenotype. We found that higher levels of miR-1915 improved capacity of tARPCs to differentiate into adipocyte-like cells leading to a larger number of oil-red positive cells after 20 days of culture in differentiation medium (Figure 6A–E). Regarding epithelial differentiation, we found a great increase of cytokeratin-19 (CK-19) in renal progenitors cultured in differentiation medium following miR-1915 mimic transfection respect to controls (mock transfected cells) after 20 days of differentiation (Figure 6F–J and Figure S8A in File S1). However, no significant difference was found in ZO-1 marker expression between mimic-transfected and mock-transfected tARPCs after 20 days of culture in epithelial differentiation medium (Figure 6L, N,O and Figure S8B in File S1). Nevertheless, we found an increase of both ZO-1 and CK-19 expression in tARPCs transfected with the miR-1915 mimic and cultured for 20 days in maintenance medium respect to mock transfected cells (Figure 6F, H, J and 6K, M, O, respectively and Figure S8 in File S1). Moreover, we found also increased levels of aquaporin 1 and aminopeptidase A transcripts in mimic-transfected tARPCs following epithelial differentiation (Figure S9 in File S1).

### miR-1225-5p Regulates TLR2 Expression in tARPCs

**Figure 7:**
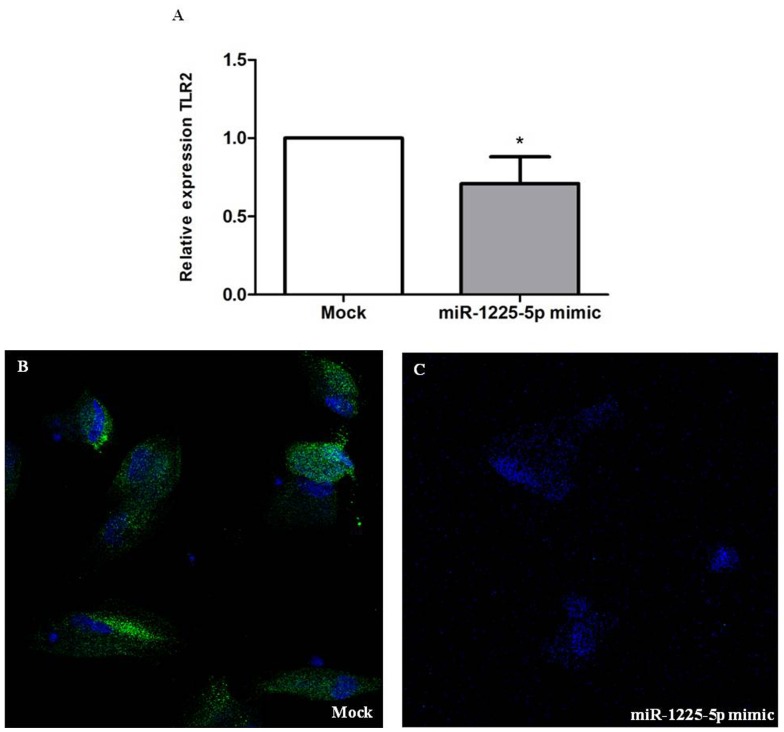
miR-1225-5p regulates TLR2 in ARPCs. (**A**) TLR2 expression levels were analyzed by real-time PCR following transfection with 25 nM miR-1225-5p mimic. Increasing the amount of miR-1225-5p within ARPCs resulted in a 1.3 fold reduction of TLR2 mRNA levels 24 hours after transfection. Expression data were normalized on the housekeeping gene β-actin. Data are representative of three independent experiments (means ± SEM), *p<0.01. (**B–C**) TLR2 protein expression after transfection with 50 nM miR-1225-5p mimic. A strong reduction of TLR2 in ARPC was found after 3 days from transfection with miR-1225-5p mimic, as shown by immunofluorescence staining. To-pro-3 counterstains nuclei (blue). Original view X63.

Our bioinformatics analysis identified a putative miR-1225-5p binding element in the 3′ UTR of TLR2 transcript, a key gene of damage signalling found up-regulated in ARPCs and that can give rise to repair processes in acute renal tubular damage [7], [16]. To examine whether miR-1225-5p downregulation led to TLR2 overexpression in tARPCs, we increased the miRNA levels transfecting renal progenitors with the miR-1225-5p mimic to simulate the higher levels of this miRNA in RPTECs. We found that the transfection with 25 nM miR-1225-5p mimic led, after 24 hours, to a reduction of 1.3 fold of the TLR2 mRNA expression (p<0.01, Figure 7A). Moreover, the increase of the miR-1225-5p levels (50 nM) completely prevented the TLR2 protein expression, compared to mock-transfected cells, 3 days after transfection (Figure 7B–C). All together these results show that, in tARPCs, TLR2 mRNA is regulated by miR-1225-5p.

## Discussion

miRNAs may be one of the key regulators of the stem cell identity, including self-renewal and cell fate decisions, thanks to their ability to simultaneously regulate many targets that provides a means for coordinated control of gene action [10]–[14]. Here we found distinct sets of miRNAs that were specifically expressed both in tARPCs and gARPCs. Among them, we found some miRNAs whose target genes overlapped with genes that were modulated in comparison to RPTECs in our previous works and whose expression resulted inverse respect to modulated genes [7]–[8], [16]. Some of these miRNAs were predicted to target genes involved in the regulation of WNT/B-catenin signals, such as FZD5, SLC6A6, and HOXA9 (Figure S2 and S3 in File S1).

In stem cells WNT signals are transduced to the canonical pathway for cell fate determination and to the noncanonical pathway for control of cell movement and tissue polarity. Target genes of WNT signaling cascades are determined in a context-dependent manner due to expression profile of transcription factors and epigenetic status [17]–[18]. Canonical WNT signals are transduced through Frizzled family receptors and LRP5/LRP6 coreceptor to the β-catenin signaling cascade. WNT, fibroblast growth factor (FGF), Notch, Hedgehog, and transforming growth factor β/bone morphogenetic protein signaling networks are implicated in the maintenance of tissue homeostasis by regulating self-renewal of normal stem cells as well as proliferation or differentiation of progenitor (transit-amplifying) cells, regulating the balance between stem and progenitor cells [17]–[18]. Specifically, Fzd5 can regulate WNTs, interpreting WNT signals during embryonic mesoderm and neural induction [19]–[20]. Moreover, it is known that HOXA9 shows positive regulation of genes in the Wnt pathway important for hematopoietic stem cell self-renewal [21].

Among many miRNAs modulated in ARPCs, we focused in particular on two of them, the miR-1915 and miR-1225-5p, that were interesting since together were predicted to target several markers typical ARPCs, such as CD133, PAX2, PAX-8, CD44 [1], [4]–[5], [7] and several genes found over-expressed in ARPCs, such as TLR2, inhibin, cyclin D1, BMP receptor 2, IL-8 and MCP-1 [7]–[8], [16]. We demonstrated that in renal progenitors the expression of both the stem cell markers CD133 and PAX2 depends, at least in part, on lower miR-1915 levels.

CD133, a pentaspan membrane glycoprotein, is a marker for adult stem cells in various tissues and tumor types. Stem cell specificity is maintained by tight regulation of CD133 expression at both transcriptional and post-translational levels but very little is known about its regulation and its molecular function [22]–[24].

However, PAX2, a transcription factor critical for kidney development [25] is another important marker of ARPCs that is also re-expressed in tubular cells damaged by renal toxins [26]–[27].

Moreover it has been shown that Activin/Inhibin A signaling can regulate the expression of PAX2 and can induce an immature phenotype in tubular cells [28].

We found that the increase of levels of miR-1915 improved capacity of tARPCs to differentiate into adipocyte-like and epithelial-like cells, suggesting that the down-regulation of CD133 and PAX2 expression led renal progenitors to be more prone to differentiation.

Another important key gene of ARPCs is TLR2 since the engagement of the receptor that it codifies can activate renal progenitors leading to increase secretion of IL-6, IL-8 and MCP-1 chemokines and enhancing differentiation capacity of renal stem/progenitor cells [7]. Moreover, the TLR2 plays an important role in the acute tubular cell injury: it can work as a sensor of the damage and drive tARPCs to repair damaged RPTECs by secretion of Inhibin-A and microvesicle-shuttled Decorin and CyclinD1 [16]. We showed that the low levels of miR-1225-5p in tARPCs were responsible for high TLR2 expression and found that the same miRNA was predicted to regulate other important genes found overexpressed in ARPCs, such as PAX-8, IL-8, BMPR2, IGF1, Inhibin-A, CyclinD1 and WNT1 [7]–[8], [16].

Therefore, together, the miR-1915 and the miR-1225-5p seem to regulate important traits of ARPCs: the stemness and the repair capacity. Their relevance was supported by the bioinformatic analyses showing that these two miRNAs have multiple target genes that are all highly interconnected and that were all found up-regulated in renal progenitors.

Further studies will be necessary to determine whether the modulation of these two miRNAs could enhance, in vivo, the regenerative capacity of ARPCs for therapeutic purposes.

## Materials and Methods

### Renal Tissue-derived Primary Cell Cultures

Fresh human renal cortical tissue was harvested from patients diagnosed with renal carcinoma. All patients gave signed consent for the use of their tissue for research purposes at the time of radical nephrectomy. The study was carried out according to the principles of the Declaration of Helsinki and was approved by our institutional ethics review board (Independent Ethical Committee of Policlinic Hospital of Bari). Portions of normal-appearing cortex were isolated surgically and examined histologically to exclude presence of carcinoma. Isolation of renal fractions was achieved as reported elsewhere [7]–[8], [15]–[16], recovering both glomerular and tubular fractions. Briefly, cortex renal fractions were dissected by the passage through a graded series of meshes steel sieves to remove the fibrous component. The cellular fraction was then passed through a 120-mesh sieve to isolate the capsulated glomeruli from the tubular fraction. After several washes, the two isolated fractions were cultured separately in EGM-MV medium (Lonza) supplemented with 20% FBS (Sigma-Aldrich). After 4–5 days, cultures were washed twice with Hanks’ buffer to remove non-adherent cells and after about 1 week in culture, cell viability and number were checked. CD133-positive cells were then isolated by magnetic cell separation technology (MACS) by means of CD133Ab-conjugated magnetic microbeads (Miltenyi Biotec). The eluted cells were resuspended and maintained in EGM-MV medium supplemented with 20% FBS and incubated at 37°C with 5.0% CO2. After cell expansion, ARPC markers were checked by cytofluorimetric determination and by cell immunofluorescence microscopy. Cytofluorimetric analysis was performed using a Partec Flow-Max cytofluorimeter (Partec). Each determination was performed on 10^5^ cells. The following antibodies were used: PE-conjugated anti-CD133/2 (293C3), FITC-conjugated anti-CD34, and FITC-conjugated anti-CD45 (all from Miltenyi Biotec); FITC-conjugated anti-CD105 and FITC-conjugated anti-CD24 (all from Serotec); and FITC-conjugated anti-CD44 (from Instrumentation Laboratory, Milan, Italy). FITC-conjugated mouse IgG1 (Serotec) was used as an isotype control. In all cytofluorimetric determinations performed using Miltenyi antibodies, nonspecific sites were blocked with the FcR blocking reagent (Miltenyi Biotec). Immunofluorescence experiments were performed using the following primary antibodies: mouse anti-human CD133/1 mAb (clone AC133; Miltenyi Biotec), mouse antihuman CD133/2 mAb (Miltenyi Biotec), rabbit anti-human PAX2 pAb (Covance, Princeton, NJ), mouse anti-human CD105 mAb (Abcam), mouse anti-human CD24 mAb (Dako), mouse anti-human CD44 mAb (Chemicon), mouse anti-human Bmi1 mAb (Upstate Biotechnology), rabbit anti-human Oct-4 pAb (Abcam). The following secondary antibodies were used: Alexa Fluor 555 goat anti-mouse IgG, Alexa Fluor 488 goat anti-rabbit IgG, and Alexa Fluor 488 goat anti-mouse IgG1 (all from Molecular Probes).

### Other Cell Cultures

MSCs and RPTECs were purchased from Lonza and maintained in the recommended medium, REGM and MSCGM, respectively (Lonza). Primary RPTECs stain positive for gamma-glutamyl transpeptidase (g-GTP) and for alkaline phosphatase and can form tubules on matrigel.

### miRNA Isolation

Total RNA was extracted from a minimum of 10^6^ cells/line using the miRNeasy Mini kit (Qiagen) according to the manufacturer’s protocol. DNase treatment was carried out to remove any contaminating DNA (RNase-Free DNase Set, Qiagen).

Total RNA, including small RNA fractions, was then eluted in RNase-free water. The RNA concentration was determined with NanoDrop Spectrophotometer (Nanodrop Technologies). The miRNAs quality was assessed by using Agilent 2100 Bioanalyzer (Agilent Technologies), and only samples with RNA integrity number >8.5 were used.

### miRNA Microarray

miRNA microarray analysis was performed using the Agilent Human miRNA Microarrays V3, which were based on Sanger miRBase release 16.0, according to the manufacturer's protocol. Briefly, 600 ng of total RNA isolated from each cell lines were first dephosphorylated with a calf intestine alkaline phosphatase treatment for 30 min. at 37°C before labeling. Samples were diluted with DMSO, denatured for 10 min. at 100°C and labeled using pCp-Cy3 in T4 RNA ligation buffer.

The labeled RNA was hybridized, washed, stained and scanned with Agilent microarray scanner (G2565BA, Agilent). Microarray data analysis was performed using Agilent Feature Extraction Software.

Microarray data are available under accession number GSE42391 at the Gene Expression Omnibus (GEO, http://www.ncbi.nlm.nih.gov/geo/).

### Statistical Analysis and Bioinformatics

For microarray analysis, the raw expression signals were log-transformed, normalized and filtered according to the median corrected of signal of all the miRNAs with an intensities >100 (which is considered as expressed) which resulted in the selection of 327 miRNAs out of the original 1205 miRNA set. miRNAs displaying differential expression between different cell lines were detected using a two-sample t-test. Probe sets were sorted after significant P-value and were adjusted to account for multiple testing using the FDR method of Benjamini-Hochberg. To determine the features that were differentially expressed between two types of cells with a stronger statistic, we applied a filter with FDR<0.01 and a fold change >2. Two-dimensional hierarchical clustering and PCA were performed using Genespring software (Agilent Technologies). To assess biological relationships among genes, we used the Ingenuity Pathway Analysis software (IPA, Ingenuity System; http://www.ingenuity. com). IPA computes a score for each network according to the fit of the set of supplied focus miRNAs/genes. These scores indicate the likelihood of focus genes to belong to a network versus those obtained by chance. In IPA, the Expression Pairing tool was then applied to display the microRNA-mRNA pairs, and to filter down the pairs with inverse expression relationships.

miRNA targets were predicted by means of miRBase 17.0 (http://microrna.sanger.ac.uk) [29], TargetScan 5.2 (http://www.targetscan.org/) [30], PicTar (http://pictar.org) [31] and RNA22 1.0 (http://cm.jefferson.edu/rna22v1.0) [32] algorithms. The algorithms interrogated the databases by searching for the presence of conserved 8mer and 7mer sites that match the seed region of each miRNA at 3'-UTR, 5'-UTR or coding sequences. The scanning algorithms were based on sequence complementarity between the mature miRNA and the target site, binding energy of the miRNA–target duplex, and evolutionary conservation of the target site sequence and target position in aligned homologous genes. Potential targets were chosen overlapping results from the four algorithms and selecting gene targets predicted by at least 2 of them and based on a score cutoff computed by a weighted sum of a number of sequence and context features of the predicted miRNA:mRNA duplex. Bioinformatic analysis to study the potential binding sites of miRNAs in CD133 target mRNA was carried out by the RNAhybrid (http://bibiserv.techfak.uni-bielefeld.de) [33]. In general, the program finds the energetically most favorable hybridizations of a small RNA to a large RNA by a thorough statistical analysis of minimum free energies (MFEs).

We evaluated statistical significance with the Student’s *t* test for comparisons between 2 mean values. Pearson’s correlation test was used to study continuous variables. All values were expressed as the mean±SD of data obtained from at least three independent experiments. Results were considered statistically significant at p<0.05.

### Quantitative RT-PCR

Total RNA, including small RNA fractions, was reverse transcribed with miScript Reverse Transcription Kit (Qiagen) following the manufacturer’s instructions.

The real-time reverse-transcription polymerase chain reaction (RT PCR) for the quantification of miRNAs was carried out with miScript Primer Assays and miScript SYBR Green PCR Kit from Qiagen. Real-time PCR amplification reactions were performed in triplicate in 25 µl of final volume via SYBR Green chemistry on iCycler (Bio-Rad).

Normalization was performed with a small nucleolar RNA U6 endogenous control. Comparative real-time PCR was performed in triplicate, including no-template controls. Relative expression was calculated using the 2^−ΔCt^ method.

For CD133, PAX2 and TLR2 expression analysis, total RNA was reverse transcribed with QuantiTect Reverse Transcription Kit (Qiagen) following the manufacturer’s instructions. Quantitative RT-PCR amplification reactions were performed in triplicate in 25 µl final volumes using SYBR Green chemistry on an iCycler. Quantitative RT-PCR was performed using the QuantiTect Primer Assay and the QuantiFast SYBR Green PCR mix (Qiagen). Genes were amplified according to the manufacturer’s directions. The β-actin gene amplification was used as a reference standard to normalize the target signal. Relative expression was calculated using the 2^−ΔCt^ method.

### miR-1915 and miR-1225-5p Mimic Transfection

For each transfection, tARPCs and gARPCs were cultured at 1*10^5^ cells per well in a 6-well plate with EGM-MV with 20% FBS at 37°C in 5% CO_2_. The transfection of miRNA mimic was carried out using TransIT-TKO Transfection Reagent (Mirus) in accordance with manufacturer's procedure. All miRNA mimics were purchased from Dharmacon (Thermo Fisher Scientific). In transfection experiments a mock-transfection control was performed by putting cells through the transfection procedure without adding miRNA. The validated nonsilencing siRNA sequence AllStars Negative Control siRNA (50 nM, Qiagen) and the Syn-hsa-miR-1 miScript miRNA Mimic (25 nM, Qiagen) were used as negative control and positive control, respectively, for the setup of miRNA mimic transfections. Each transfection experiment was done in triplicate. After transfection, cells were incubated for 24 hours for total RNA extraction. For the protein analysis, cells were transfected twice at a distance if 3 days and the protein expression was assayed 6 days after the first transfection.

### Flow Cytometric Analysis

The CD133 protein surface expression, after transfection, was determined by flow cytometric analysis. ARPCs were washed and resuspended in FACS buffer and incubated with PE-conjugated anti-CD133/2 (293C3) (Miltenyi Biotec). Non-specific sites were blocked with the FcR blocking reagent (Miltenyi Biotec). Cells were analyzed on a ‘‘EPICS XL’’ Flow Cytometer (Beckman Coulter). The area of positivity was determined using an isotype-matched mAb, a total of 10^4^ events for each sample were acquired.

### Western Blot Analysis

The amount of PAX2, after transfection, was determined by Western blotting analysis. Total protein extracts were prepared with RIPA lysis buffer containing 150 mM NaCl, 20 mM Tris-HCl (pH 7.4), 5 mM EDTA, 1.5% NonidetP-40, 1 mM sodium orthovanadate, plus proteinase inhibitors. The protein concentration was determined by the Bradford assay (BioRad). 80 µg of each protein lysate was separated on a 10% SDS-PAGE and transferred to polyvinyldene difluoride (PVDF) membrane (Millipore). The membranes were incubated in 5% non-fat milk powder diluted in PBS containing 0.1% Tween-20 (T-PBS) for 2 h at room temperature (RT) and probed with a rabbit polyclonal anti–PAX2 antibody (Novus Biologicals) in blocking buffer overnight at 4°C. Finally, membranes were incubated with secondary antibody of horseradish peroxidase conjugated goat anti-rabbit IgG (Santa Cruz). Immunocomplexes were detected with ECL method (GE Healthcare). The same membranes were stripped and reprobed with anti- β-actin monoclonal antibody (Sigma).

Images of autoradiography were acquired using a scanner EPSON Perfection 2580 Photo (EPSON) and quantified by Image J 1.34 Software (http://rsb.info.nih.gov/ij/). Ratio between intensities of PAX2 and β-actin bands was used to normalize PAX2 expression in each sample.

### Cell Immunofluorescence and Confocal Laser Scanning Microscopy

The expression of TLR2, after transfection, was evaluated by indirect immunofluorescence and confocal microscopic analysis on ARPCs placed on a cover slip and fixed in 4% paraformaldehyde. The cells were blocked for 1 h (BSA in PBS, pH 7.4) and then incubated with a mouse anti-human TLR2 mAb (Hycult Biotechnology) and a mouse anti-human CD133 mAb (Miltenyi Biotec) overnight at 4°C. The immune complexes were identified after the incubation of the cells with the specific secondary antibodies (Alexa Flour 488 and Alexa Fluor 555 goat anti-mouse IgG, respectively) for 1 h at room temperature. The cells were washed in PBS after each step, counterstained with To-pro-3 (Molecular Probes), mounted in Gel/Mount (Biomeda), and sealed with nail varnish. Negative controls were obtained by incubating cells with the blocking solution and then omitting the primary antibody. The stained cells were viewed under the Leica TCS SP2 (Leica, Wetzlar, Germany) confocal laser-scanning microscope using x40 and x63 objective lenses. Fluorescence levels were quantified and expressed as area fraction (%).

### Differentiation

To evaluate the effects of the miR-1915 on ARPCs differentiation, the ARPCs were transfected with 50 nM miR-1915 mimic (Dharmacon) at the first day of differentiation and every 7 days. The undifferentiated and untransfected cells were used as negative controls.

The extrarenal differentiation experiments were performed using the adipogenic induction/maintenance medium and osteogenic differentiation medium (Lonza), according to the manufacturer’s instructions. For adipogenic differentiation, 3 cycles of induction/maintenance were performed. Each cycle consisted of culturing ARPCs in induction medium for 3 d and in maintenance medium for 1–3 d. After 3 complete cycles, the cells were cultured for seven more days in adipogenic maintenance medium, replacing the medium every 2–3 d. At the end of the treatment the lipid vacuoles were stained with Oil red-O (Sigma). Briefly, cells were washed with PBS, fixed with 3.7% paraformaldehyde for 15 min, rinsed with PBS and incubated with Oil red-O for 30 min.

Epithelial differentiation was obtained by culturing ARPCs in commercially available REGM medium (Lonza) supplemented with 50 ng/ml hepatocyte growth factor (Sigma) for 20 days. Tubular cells were identified by the expression of CK-19 and ZO-1 in immunofluorescence using rabbit anti-human ZO-1 polyclonal Ab (Santa Cruz Biotechnology) and rabbit anti-human CK-19 mAb (Novus Biologicals).

## Supporting Information

File S1 **Figure S1.** **Venn diagram on the common and distinct miRNAs modulated in tARPCs, gARPCs and MSCs respect to RPTECs.Figure S2. Bioinformatics analysis by Ingenuity Pathway Analysis software (IPA).** (A) Significant pathways in which miRNAs modulated in ARPCs were involved. B) Significant biological processes in which miRNAs modulated in ARPCs were involved. **Figure S3. Functional analysis of the top modulated miRNAs identified by microarray.** The network was algorithmically constructed by Ingenuity Pathway Analysis (IPA) software on the basis of the functional and biological connectivity of miRNAs and genes. The network is graphically represented as nodes (miRNAs or genes) and edges (the biological relationship between miRNAs/genes). Merging miRNAs with ARPC modulated genes, a significant network was identified including modulated genes involved in the WNT/B-catenin signalling, as FZD5. Red and green shaded nodes represent up- and down-regulated genes/miRNAs, respectively; empty nodes represent modulated miRNAs or genes that IPA automatically includes because they are biologically linked to our genes/miRNAs based on the evidence in the literature. **Figure S4. Functional analysis of the top modulated miRNAs identified by microarray.** The network was algorithmically constructed by Ingenuity Pathway Analysis (IPA) software on the basis of the functional and biological connectivity of miRNAs and genes. The network is graphically represented as nodes (miRNAs or genes) and edges (the biological relationship between miRNAs/genes). Merging miRNAs with ARPC modulated genes, a significant network was identified including modulated genes involved in the Frizzled signalling, as SLC6A6 and HOXA9. Red and green shaded nodes represent up- and down-regulated genes/miRNAs, respectively; empty nodes represent modulated miRNAs or genes that IPA automatically includes because they are biologically linked to our genes based on the evidence in the literature. **Figure S5. Bioinformatics analysis by Ingenuity Pathway Analysis software (IPA).** (A) Significant pathways in which miRNAs modulated in MSCs were involved. B) Significant biological processes in which miRNAs modulated in MSCs were involved. **Figure S6. Functional analysis of the top modulated miRNAs identified by microarray.** The network was algorithmically constructed by Ingenuity Pathway Analysis (IPA) software on the basis of the functional and biological connectivity of miRNAs and genes. The network is graphically represented as nodes (miRNAs or genes) and edges (the biological relationship between miRNAs/genes). Merging miRNAs with MSC modulated genes, a significant network was identified including modulated genes involved in cellular movement, cell morphology and cell-to-cell signalling and interaction. Red and green shaded nodes represent up- and down-regulated genes/miRNAs, respectively; empty nodes represent modulated miRNAs or genes that IPA automatically includes because they are biologically linked to our genes/miRNAs based on the evidence in the literature. **Figure S7. Functional analysis of the top modulated miRNAs identified by microarray.** The network was algorithmically constructed by Ingenuity Pathway Analysis (IPA) software on the basis of the functional and biological connectivity of miRNAs and genes. The network is graphically represented as nodes (miRNAs or genes) and edges (the biological relationship between miRNAs/genes). Merging miRNAs with MSC modulated genes, a significant network was identified including modulated genes involved in cellular movement and tissue morphology. Red and green shaded nodes represent up- and down-regulated genes/miRNAs, respectively; empty nodes represent modulated miRNAs or genes that IPA automatically includes because they are biologically linked to our genes/miRNAs based on the evidence in the literature. **Figure S8. Effect of miR-1915 on tARPC epithelial differentiation.** Western blot analysis show CK-19 (A) e ZO-1 (B) in tARPCs cultured in maintenance and differentiation medium, transfected and not-transfected with miR-1915 mimic (50 nM), respectively. Increased levels of CK-19 (A) were observed in tARPCs cultured in differentiation medium following miR-1915 mimic transfection after 20 days of differentiation. (B) No significant differences in ZO-1protein expression were observed in tARPCs transfected with miR-1915 mimic in differentiation medium. β-actin was used as endogenous control. Mock indicates mock-transfected cells going through the transfection processes without addition of mimic miRNA. Data are representative of three independent experiments (means ± SEM). *p<0.01. **Figure S9. Effect of miR-1915 on tARPC epithelial differentiation.** Assessment by quantitative RT-PCR of mRNA levels for tubular markers Glutamyl Aminopeptidase (aminopeptidase A, ENPEP) (A) and Aquaporin 1 (AQP1) (B) after 20 day of culture in tubular differentiation medium. Levels of aquaporin 1 and aminopeptidase A transcripts increased in miR-1915 mimic-transfected tARPCs. Mock indicates mock-transfected cells going through the transfection processes without addition of mimic miRNA. Data are representative of three independent experiments (means ± SEM). *p<0.01.
(PDF)

Table S1 **Predicted target of miRNAs differentially expressed by ARPCs versus RPTECs with a fold-change ≥2.** The genes reported in the table represents only genes of great interest for stem cell biology and were identified by the algorithms for the identification of microRNA targets that are present in miRBase 17.0, TargetScan 5.2, PicTar and RNA22 1.0 with high alignment score output. The green boxes represent the down-regulated miRNAs, instead red boxes show the up-regulated miRNAs. The blue boxes indicate the target genes predicted by at least 2 of 4 bioinformatic algorithms. The numbers in blue boxes represent the binding energy of the miRNA–target duplex. (see Excel file)
(XLS)

Table S2 **ARPC miRNAs and mRNAs with an inverse pairs expression relationships obtained merging miRNAs with modulated genes resulting from the gene expression profile (GEO Series Accession Number GSE8611).** miRNAs were strongly interconnected with the expressed mRNAs with an inverse pairs expression relationships. (see Excel file)
(XLS)
