# Correction: miR-1915 and miR-1225-5p Regulate the Expression of CD133, PAX2 and TLR2 in Adult Renal Progenitor Cells

**DOI:** 10.1371/journal.pone.0128258

**Published:** 2015-05-08

**Authors:** Fabio Sallustio, Grazia Serino, Vincenzo Costantino, Claudia Curci, Sharon N. Cox, Giuseppe De Palma, Francesco P. Schena

A reader noted a number of issues with Figure 1. The figure legend was:

Figure 1 panels D, E, & F (representing tubular adult renal progenitor cells) come from the same original images as panels J, K, & L (representing glomerular ARPC cells), respectively. Between E & K, there is a small overlap. Between panels D & J and F & L, respectively, the images have been rotated and there is a large overlap. Panels F and L are identical to panels in Sallustio et al. FASEB Journal (2010) (doi: 10.1096/fj.09-136481; Figure 1D, rotated) and Sallustio et al. Kidney International (2013) (doi:10.1038/ki.2012.413, Figure 1F and 1M, rotated). Panels D and J from the PLOS ONE paper are identical to Figure 1D of Sallustio et al. Kidney International (2013), though flipped (doi:10.1038/ki.2012.413).

An investigation by a Scientific Committee at the University of Bari concluded that these image duplications arose by inadvertent error, due to poor management of the image archive, and that this does not affect the results and conclusions. The authors apologize for these errors in the published article.

The authors have provided the correct image files for panels D, E, F, J, K, and L in Figure 1, showing immunofluorescence of tubular ARPCs with Oct-4 (D), PAX2 (E), BMI-1 (F), and of glomerular ARPCs with Oct-4 (J), PAX2 (K), BMI-1 (L).

In a subsequent PLOS ONE article, Figure 3 reproduced panels from Figure 1 of this article: Sciancalepore AG, Sallustio F, Girardo S, Gioia Passione L, Camposeo A, et al. (2014) A Bioartificial Renal Tubule Device Embedding Human Renal Stem/Progenitor Cells. PLOS ONE 9(1): e87496. doi:10.1371/journal.pone.0087496. A correction is also being made to that article.

**Fig. 1. Characterization of isolated glomerular and tubular ARPCs**. Cytofluorimetric and immunofluorescence analysis of tARPCs showed the expression of: CD133 (A), CD24 (B), CD44 (C), Oct-4 (D), PAX2 (E), BMI-1 (F), Cytofluorimetric and immunofluorescence analysis of gARPCs showed the expression of: CD133 (G), CD24 (H), CD44 (I), Oct-4 (J), PAX2 (K), BMI-1 (L). CD106 expression in glomerular (M) and tubular (N) ARPCs. Original view: X63.

## Supporting Information

S1 File Corrected files for Fig 1 D-F & J-L. Characterization of isolated glomerular and tubular ARPCs, showing immunofluorescence of tubular ARPCs with Oct-4 (D), PAX2 (E), BMI-1 (F), and of glomerular ARPCs with Oct-4 (J), PAX2 (K), BMI-1 (L).
(ZIP)
