# Supplementary material for: miR-1915 and miR-1225-5p Regulate the Expression of CD133, PAX2 and TLR2 in Adult Renal Progenitor Cells
Source: PLoS One. 2013 Jul 8;8(7):e68296. doi: 10.1371/journal.pone.0068296 (PMC3704645; doi:10.1371/journal.pone.0068296)
Supplement: File S1 — Figure S1. Venn diagram on the common and distinct miRNAs modulated in tARPCs, gARPCs and MSCs respect to RPTECs.Figure S2. Bioinformatics analysis by Ingenuity Pathway Analysis software (IPA). (A) Significant pathways in which miRNAs modulated in ARPCs were involved. B) Significant biological processes in which miRNAs modulated in ARPCs were involved. Figure S3. Functional analysis of the top modulated miRNAs identified by microarray. The network was algorithmically constructed by Ingenuity Pathway Analysis (IPA) software on the basis of the functional and biological connectivity of miRNAs and genes. The network is graphically represented as nodes (miRNAs or genes) and edges (the biological relationship between miRNAs/genes). Merging miRNAs with ARPC modulated genes, a significant network was identified including modulated genes involved in the WNT/B-catenin signalling, as FZD5. Red and green shaded nodes represent up- and down-regulated genes/miRNAs, respectively; empty nodes represent modulated miRNAs or genes that IPA automatically includes because they are biologically linked to our genes/miRNAs based on the evidence in the literature. Figure S4. Functional analysis of the top modulated miRNAs identified by microarray. The network was algorithmically constructed by Ingenuity Pathway Analysis (IPA) software on the basis of the functional and biological connectivity of miRNAs and genes. The network is graphically represented as nodes (miRNAs or genes) and edges (the biological relationship between miRNAs/genes). Merging miRNAs with ARPC modulated genes, a significant network was identified including modulated genes involved in the Frizzled signalling, as SLC6A6 and HOXA9. Red and green shaded nodes represent up- and down-regulated genes/miRNAs, respectively; empty nodes represent modulated miRNAs or genes that IPA automatically includes because they are biologically linked to our genes based on the evidence in the literature. Figure S5. Bioinformatics analysis by [file pone.0068296.s001.pdf]

SUPPORTING INFORMATION

miR-1915 and miR-1225-5p Regulate the Expression of CD133, PAX2 and TLR2 in Adult Renal Progenitor Cells

AUTHORS

Fabio Sallustio*[1,2], Grazia Serino*[1], Vincenzo Costantino[1], Claudia Curci[2], Sharon N. Cox[1], Giuseppe De Palma[2], Francesco P. Schena[1,2,3].

[1]Department of Emergency and Organ Transplantation, University of Bari, Bari, Italy.

[2]C.A.R.S.O. Consortium, Valenzano, Bari, Italy.

[3] Department of Science, Biological and Environmental Sciences and Technologies, University of Salento, Lecce, Italy.

[4]Schena Foundation, European Research Center for Kidney Diseases, Valenzano, Bari, Italy.

*These two authors contributed equally to this work.

SUPPLEMENTARY FIGURES

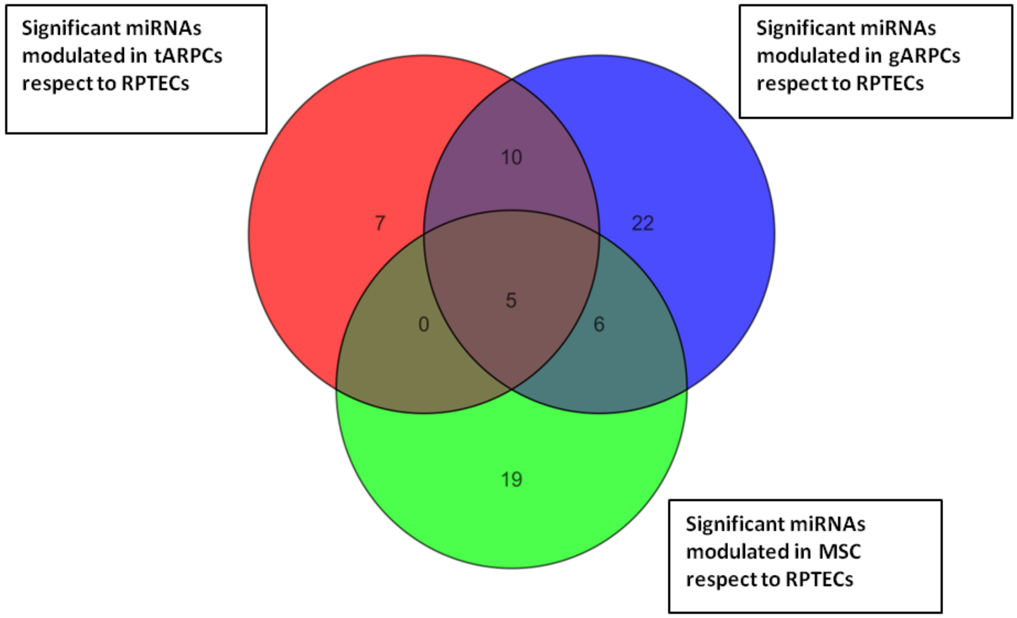

miRNAs modulated exclusively in tARPCs respect to RPTECs	miRNAs modulated exclusively in gARPCs respect to RPTECs	miRNAs modulated exclusively in MSCs respect to RPTECs	miRNAs modulated respect to RPTECs in common exclusively between tARPCs and gARPCs	miRNAs modulated respect to RPTECs in common exclusively between gARPCs and MSCs	miRNAs modulated respect to RPTECs in common between tARPCs, gARPCs and MSCs
hsa-miR-93	hsa-miR-7	hsa-let-7i	hsa-miR-130b	hsa-miR-874	hsa-miR-1225-5p
hsa-miR-1306	hsa-miR-887	hsa-miR-30a	hsa-miR-16	hsa-miR-320c	hsa-miR-193b
hsa-miR-1977	hsa-miR-625	hsa-miR-193a-5p	hsa-miR-373*	hsa-miR-1207-5p	hsa-miR-20b
hsa-miR-1226*	hsa-miR-196b	hsa-miR-30b	hsa-miR-17	hsa-miR-150*	hsa-miR-125b
hsa-miR-20a	hsa-miR-128	hsa-miR-30a*	hsa-miR-455-3p	hsa-miR-320b	hsa-miR-100
hsa-miR-15a	hsa-miR-484	hsa-miR-99a	hsa-miR-1181	hsa-miR-320d	
hsa-miR-25	hsa-miR-92a	hsa-miR-29b-1*	hsa-miR-1915		
	hsa-miR-320a	hsa-miR-30c	hsa-miR-371-5p		
	hsa-miR-7*	hsa-miR-28-5p	hsa-miR-16*		
	hsa-miR-505	hsa-let-7b	hsa-miR-15b		
	hsa-miR-296-5p	hsa-miR-140-5p			
	hsa-miR-629*	hsa-miR-29a			
	hsa-miR-663	hsa-miR-155			
	hsa-miR-134	hsa-miR-23a			
	hsa-miR-574-3p	hsa-miR-221*			
	hsa-miR-718	hsa-miR-10a			
	hsa-miR-1469	hsa-miR-140-3p			
	hsa-miR-196a	hsa-miR-30d			
	hsa-miR-197	hsa-miR-30e*			
	hsa-miR-181a*				
	hsa-miR-151-3p				
	hsa-miR-602				

Figure S1. Venn diagram on the common and distinct miRNAs modulated in tARPCs, gARPCs and MSCs respect to RPTECs.

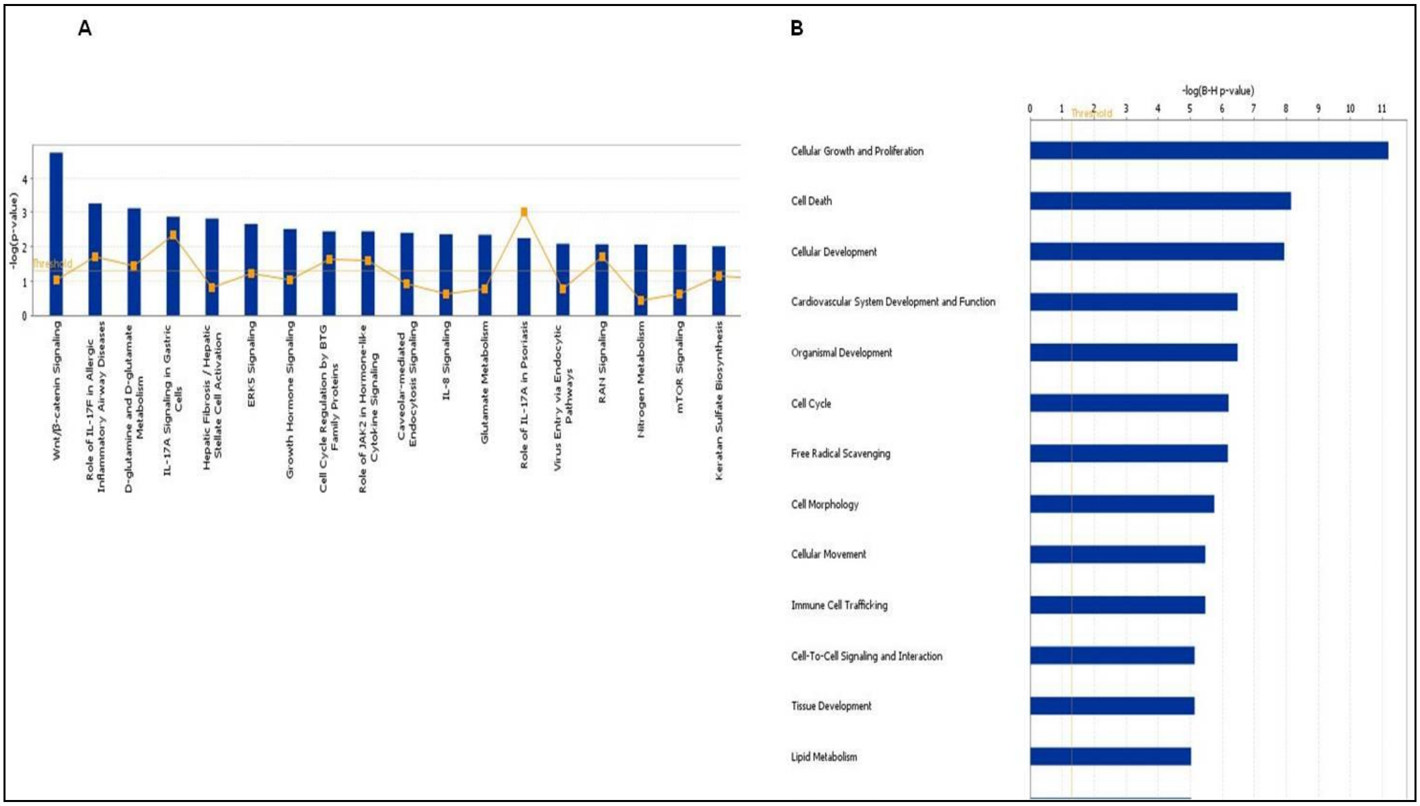

Figure S2. Bioinformatics analysis by Ingenuity Pathway Analysis software (IPA). (A) Significant pathways in which miRNAs modulated in ARPCs were involved. B) Significant biological processes in which miRNAs modulated in ARPCs were involved.

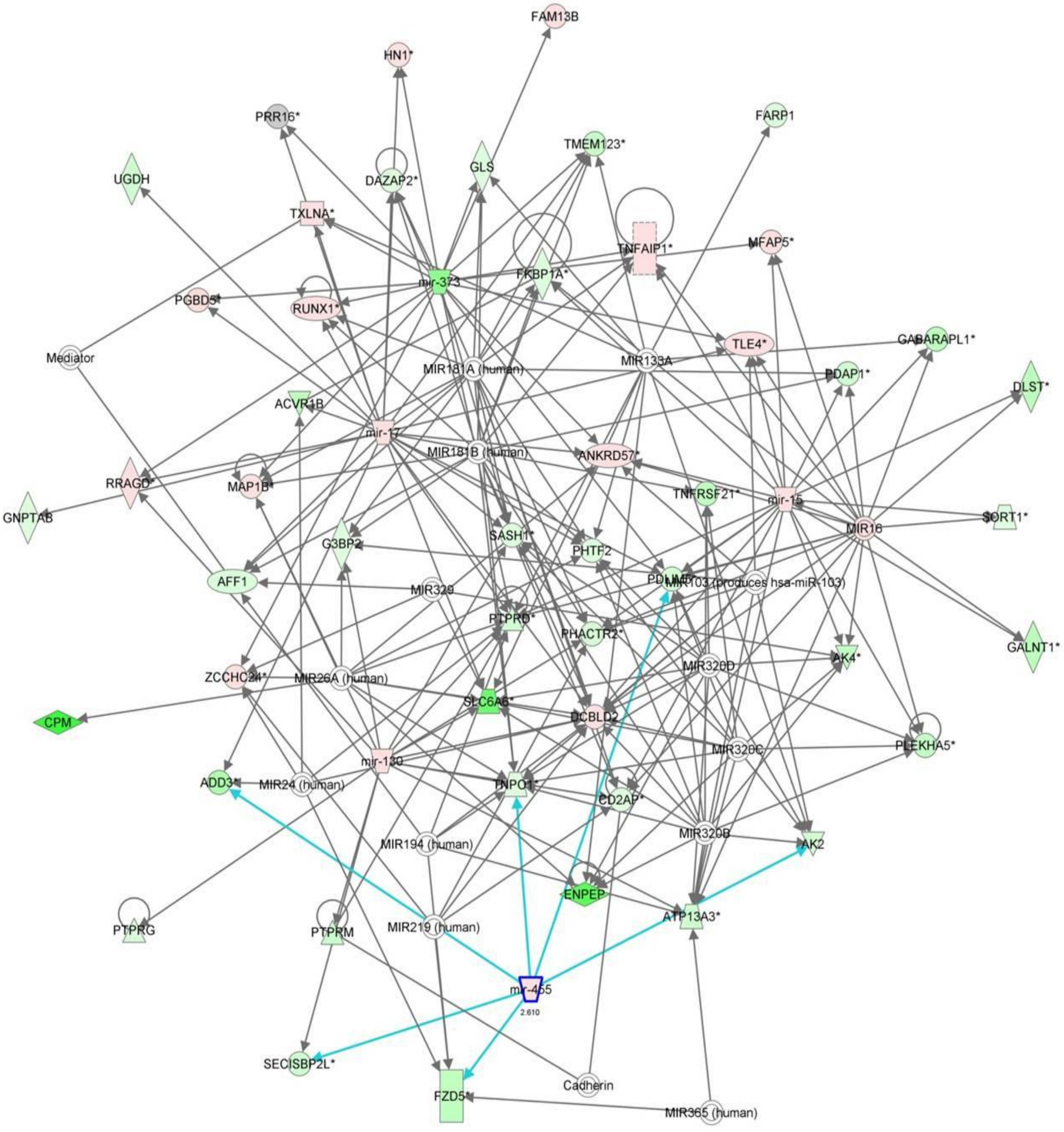

Figure S3. Functional analysis of the top modulated miRNAs identified by microarray. The network was algorithmically constructed by Ingenuity Pathway Analysis (IPA) software on the basis of the functional and biological connectivity of miRNAs and genes. The network is graphically represented as nodes (miRNAs or genes) and edges (the biological relationship between miRNAs/genes). Merging

miRNAs with ARPC modulated genes, a significant network was identified including modulated genes involved in the WNT/B-catenin signalling, as FZD5. Red and green shaded nodes represent up- and down-regulated genes/miRNAs, respectively; empty nodes represent modulated miRNAs or genes that IPA automatically includes because they are biologically linked to our genes/miRNAs based on the evidence in the literature.

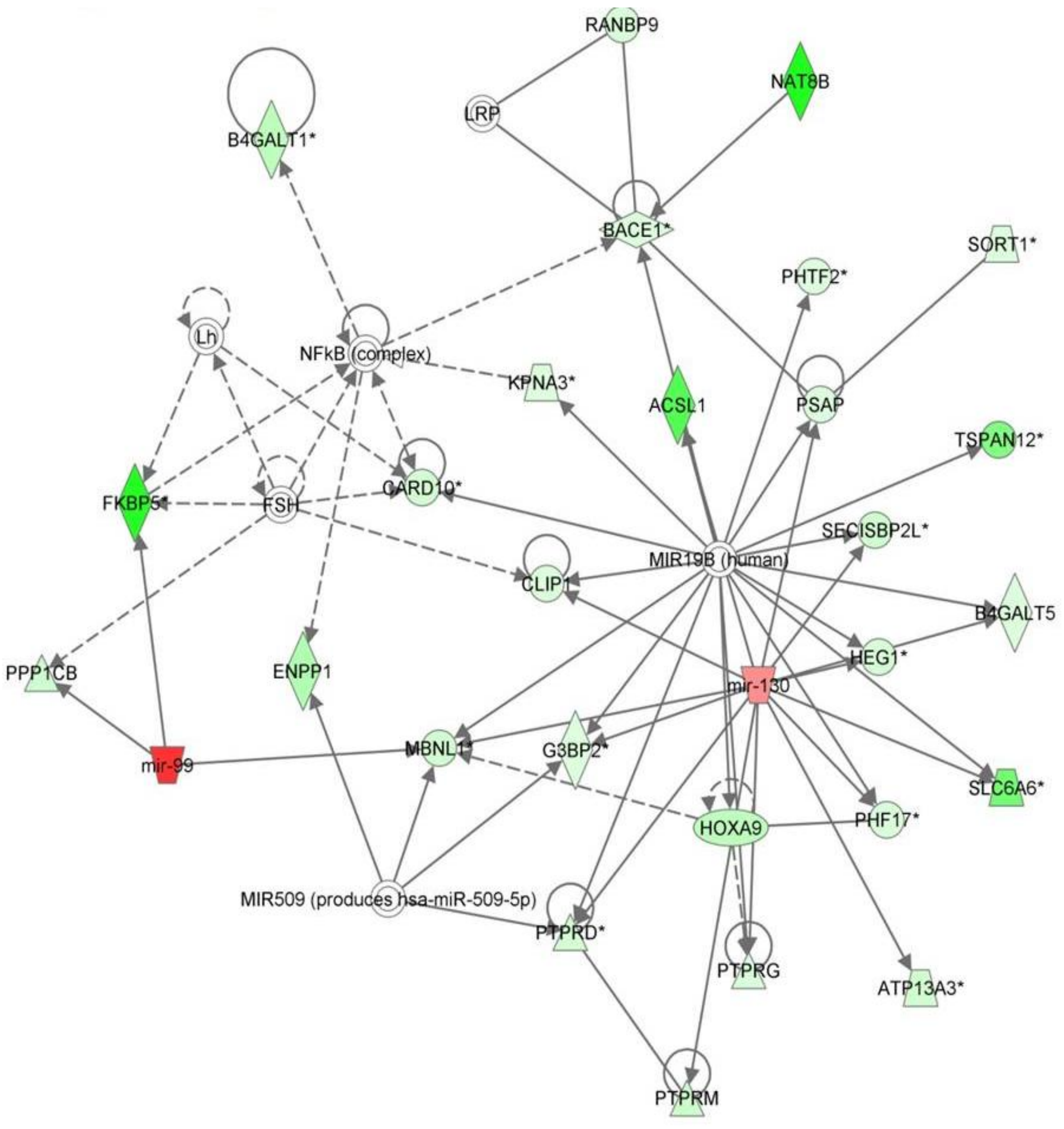

Figure S4. Functional analysis of the top modulated miRNAs identified by microarray. The network was algorithmically constructed by Ingenuity Pathway Analysis (IPA) software on the basis of the functional and biological connectivity of miRNAs and genes. The network is graphically represented as

nodes (miRNAs or genes) and edges (the biological relationship between miRNAs/genes). Merging miRNAs with ARPC modulated genes, a significant network was identified including modulated genes involved in the Frizzled signalling, as SLC6A6 and HOXA9. Red and green shaded nodes represent up- and down-regulated genes/miRNAs, respectively; empty nodes represent modulated miRNAs or genes that IPA automatically includes because they are biologically linked to our genes based on the evidence in the literature.

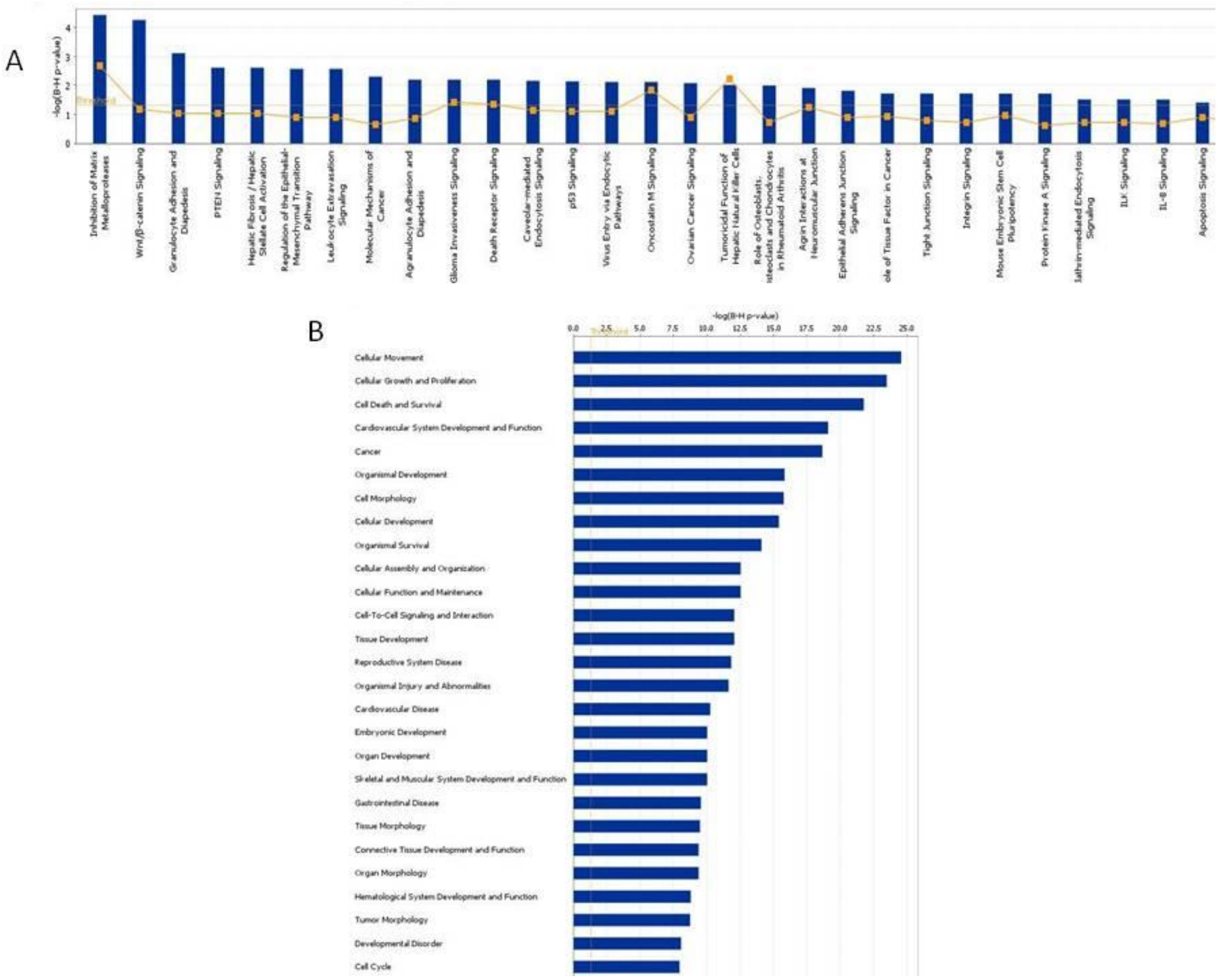

Figure S5. Bioinformatics analysis by Ingenuity Pathway Analysis software (IPA). (A) Significant pathways in which miRNAs modulated in MSCs were involved. B) Significant biological processes in which miRNAs modulated in MSCs were involved.

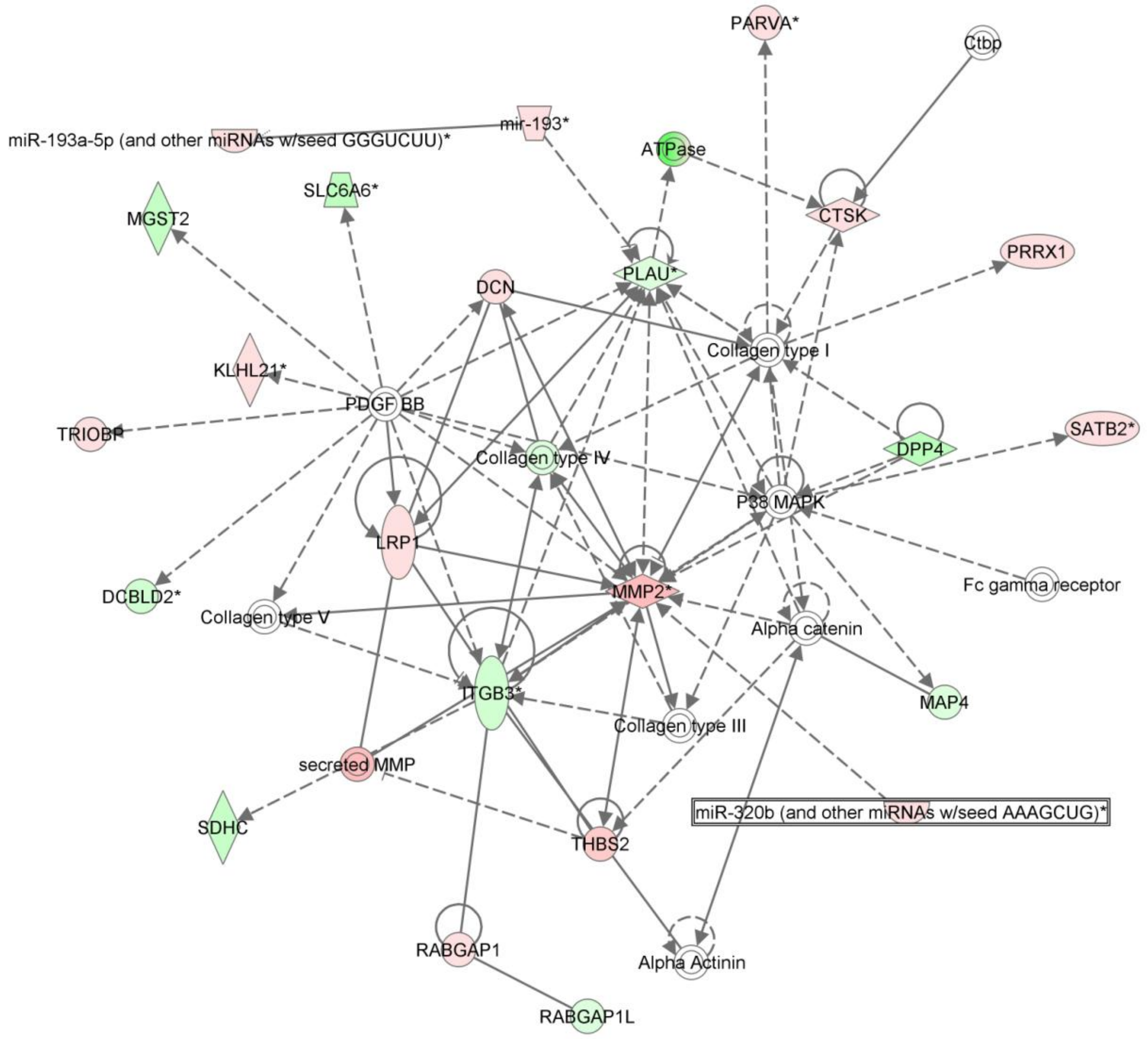

Figure S6. Functional analysis of the top modulated miRNAs identified by microarray. The network was algorithmically constructed by Ingenuity Pathway Analysis (IPA) software on the basis of the functional and biological connectivity of miRNAs and genes. The network is graphically represented as nodes (miRNAs or genes) and edges (the biological relationship between miRNAs/genes). Merging miRNAs with MSC modulated genes, a significant network was identified including modulated genes involved in cellular movement, cell morphology and cell-to-cell signalling and interaction. Red and green shaded nodes represent up- and down-regulated genes/miRNAs, respectively; empty nodes

represent modulated miRNAs or genes that IPA automatically includes because they are biologically linked to our genes/miRNAs based on the evidence in the literature.

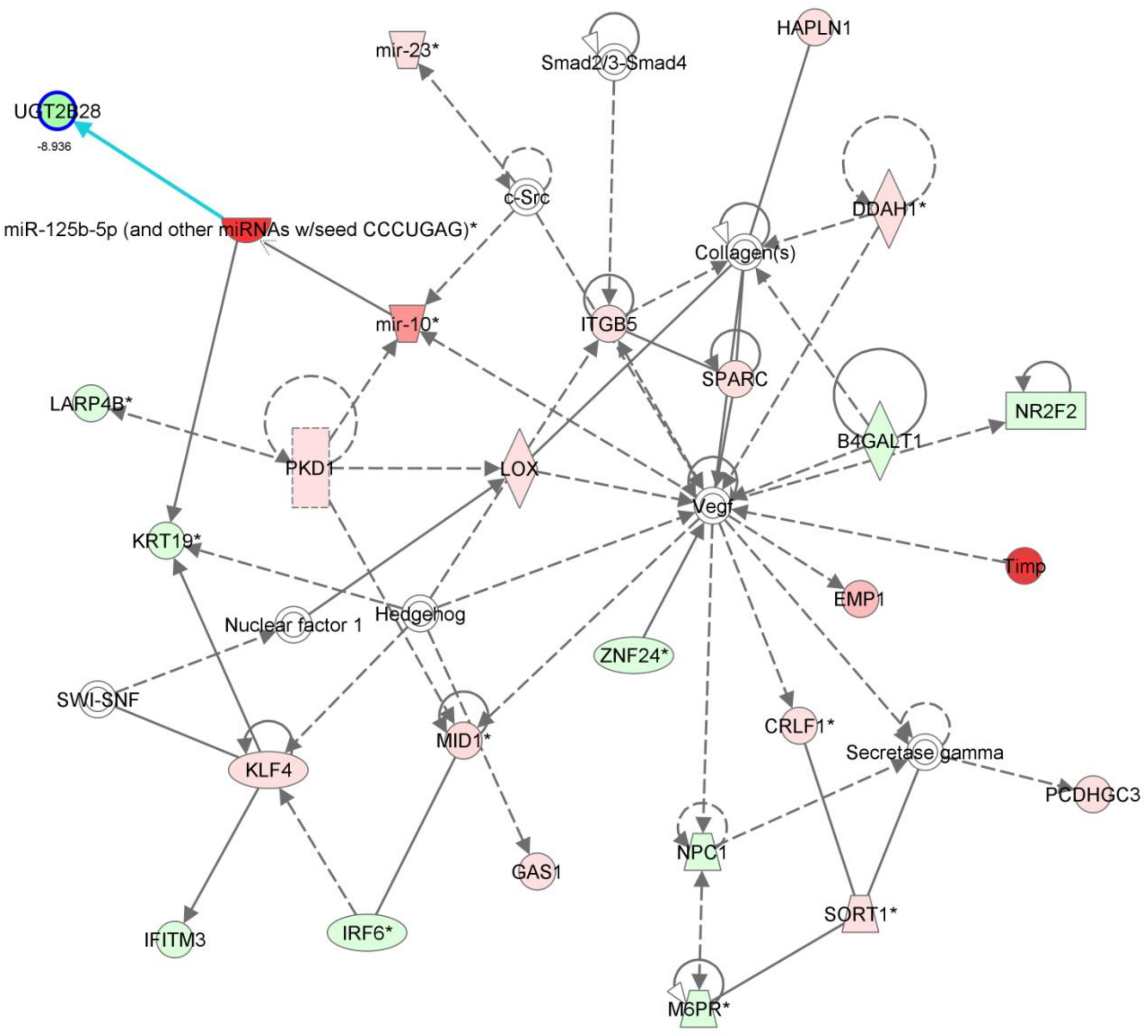

Figure S7. Functional analysis of the top modulated miRNAs identified by microarray. The network was algorithmically constructed by Ingenuity Pathway Analysis (IPA) software on the basis of the functional and biological connectivity of miRNAs and genes. The network is graphically represented as nodes (miRNAs or genes) and edges (the biological relationship between miRNAs/genes). Merging miRNAs with MSC modulated genes, a significant network was identified including modulated genes

involved in cellular movement and tissue morphology. Red and green shaded nodes represent up- and down-regulated genes/miRNAs, respectively; empty nodes represent modulated miRNAs or genes that IPA automatically includes because they are biologically linked to our genes/miRNAs based on the evidence in the literature.

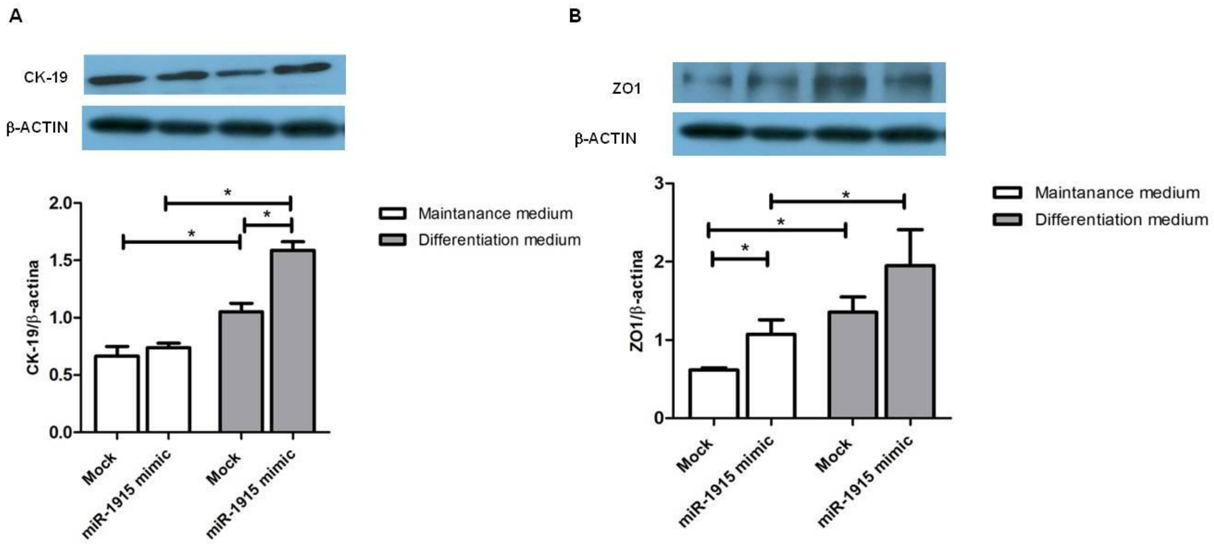

Figure S8. Effect of miR-1915 on tARPC epithelial differentiation. Western blot analysis show CK-19 (A) e ZO-1 (B) in tARPCs cultured in maintenance and differentiation medium, transfected and not-transfected with miR-1915 mimic (50 nM), respectively. Increased levels of CK-19 (A) were observed in tARPCs cultured in differentiation medium following miR-1915 mimic transfection after 20 days of differentiation. (B) No significant differences in ZO-1protein expression were observed in tARPCs transfected with miR-1915 mimic in differentiation medium. β-actin was used as endogenous control. Mock indicates mock-transfected cells going through the transfection processes without addition of mimic miRNA. Data are representative of three independent experiments (means ± SEM). *$p < 0.01$.

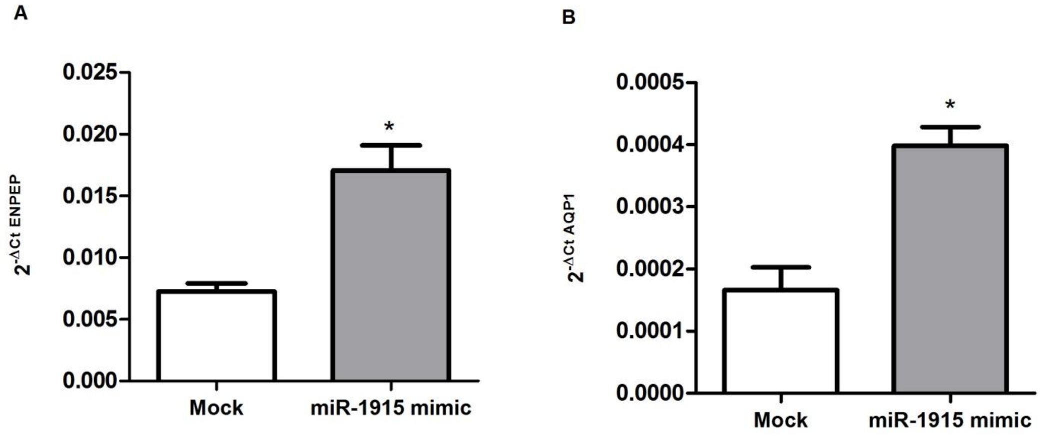

Figure S9. Effect of miR-1915 on tARPC epithelial differentiation. Assessment by quantitative RT-PCR of mRNA levels for tubular markers Glutamyl Aminopeptidase (aminopeptidase A, ENPEP) (A) and Aquaporin 1 (AQP1) (B) after 20 day of culture in tubular differentiation medium. Levels of aquaporin 1 and aminopeptidase A transcripts increased in miR-1915 mimic-transfected tARPCs. Mock indicates mock-transfected cells going through the transfection processes without addition of mimic miRNA. Data are representative of three independent experiments (means ± SEM). *p < 0.01.
