# Supplementary figures and images for: Correction: miR-1915 and miR-1225-5p Regulate the Expression of CD133, PAX2 and TLR2 in Adult Renal Progenitor Cells
Source: PLoS One. 2015 May 8;10(5):e0128258. doi: 10.1371/journal.pone.0128258 (PMC4425473; doi:10.1371/journal.pone.0128258)

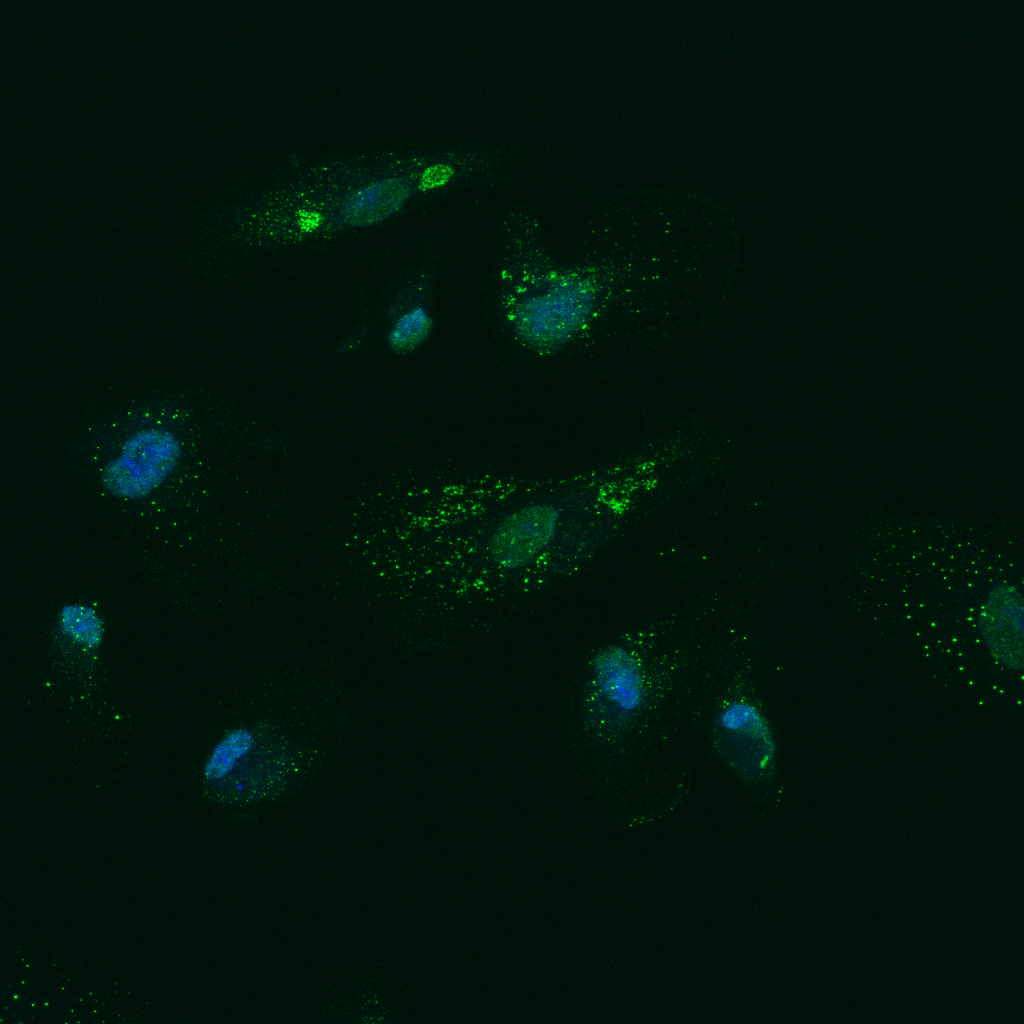

Supplement: S1 File — Characterization of isolated glomerular and tubular ARPCs, showing immunofluorescence of tubular ARPCs with Oct-4 (D), PAX2 (E), BMI-1 (F), and of glomerular ARPCs with Oct-4 (J), PAX2 (K), BMI-1 (L). (ZIP) [file pone.0128258.s001.zip › SC characterization/SC characterization/BMI/GLOM/Esp11-01-06 Stem cells_OvPjm1 BMi1glom .tif]

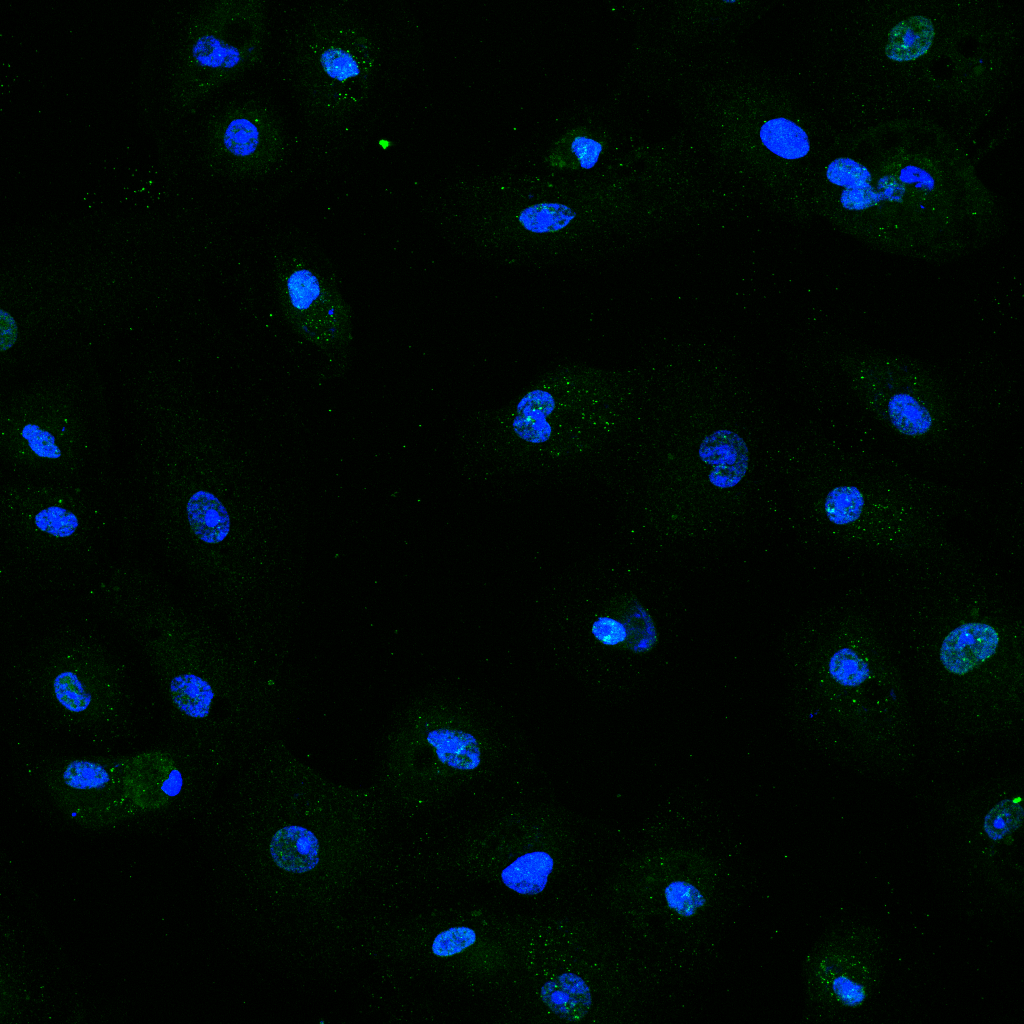

Supplement: S1 File — Characterization of isolated glomerular and tubular ARPCs, showing immunofluorescence of tubular ARPCs with Oct-4 (D), PAX2 (E), BMI-1 (F), and of glomerular ARPCs with Oct-4 (J), PAX2 (K), BMI-1 (L). (ZIP) [file pone.0128258.s001.zip › SC characterization/SC characterization/BMI/TUB/10-3-06cloni stem cells_OvPjm1 BMI1tubul.tif]

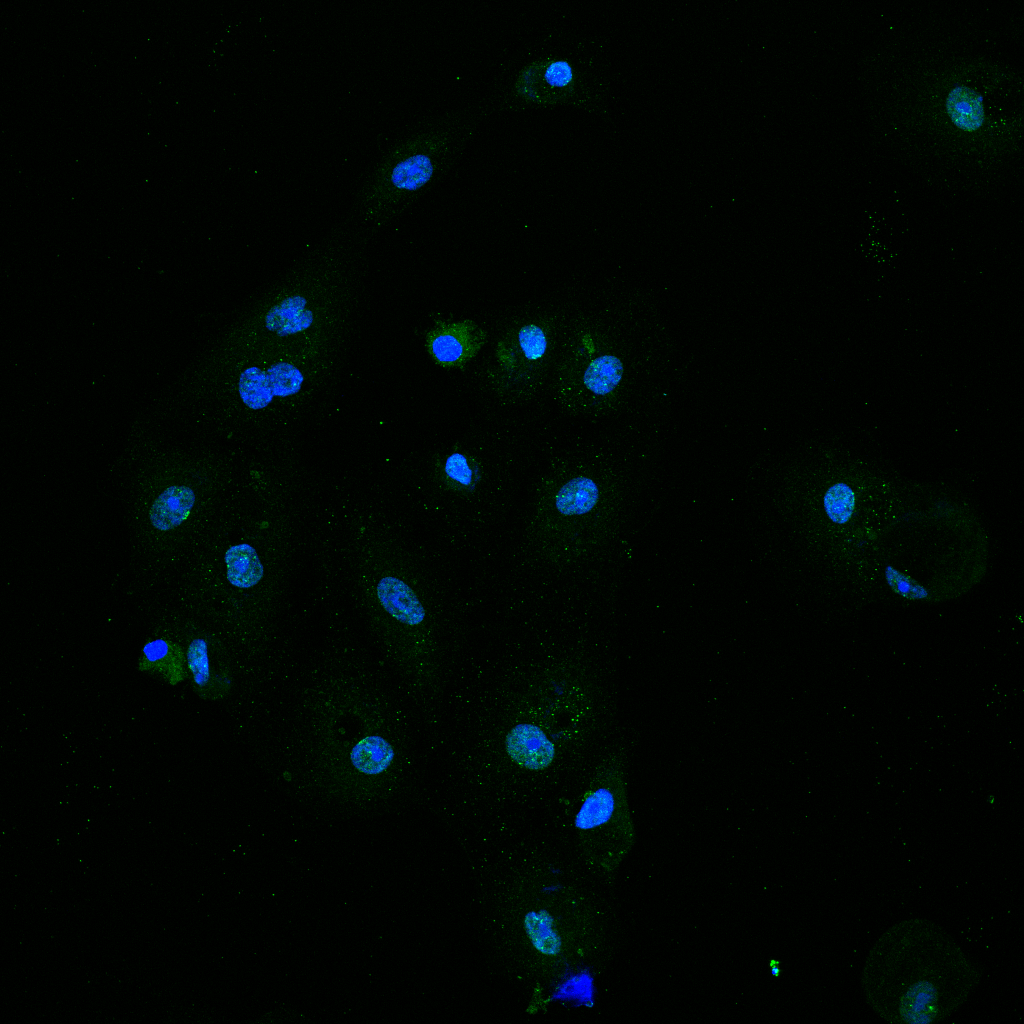

Supplement: S1 File — Characterization of isolated glomerular and tubular ARPCs, showing immunofluorescence of tubular ARPCs with Oct-4 (D), PAX2 (E), BMI-1 (F), and of glomerular ARPCs with Oct-4 (J), PAX2 (K), BMI-1 (L). (ZIP) [file pone.0128258.s001.zip › SC characterization/SC characterization/BMI/TUB/10-3-06cloni stem cells_OvPjm2 BMI1tubul.tif]

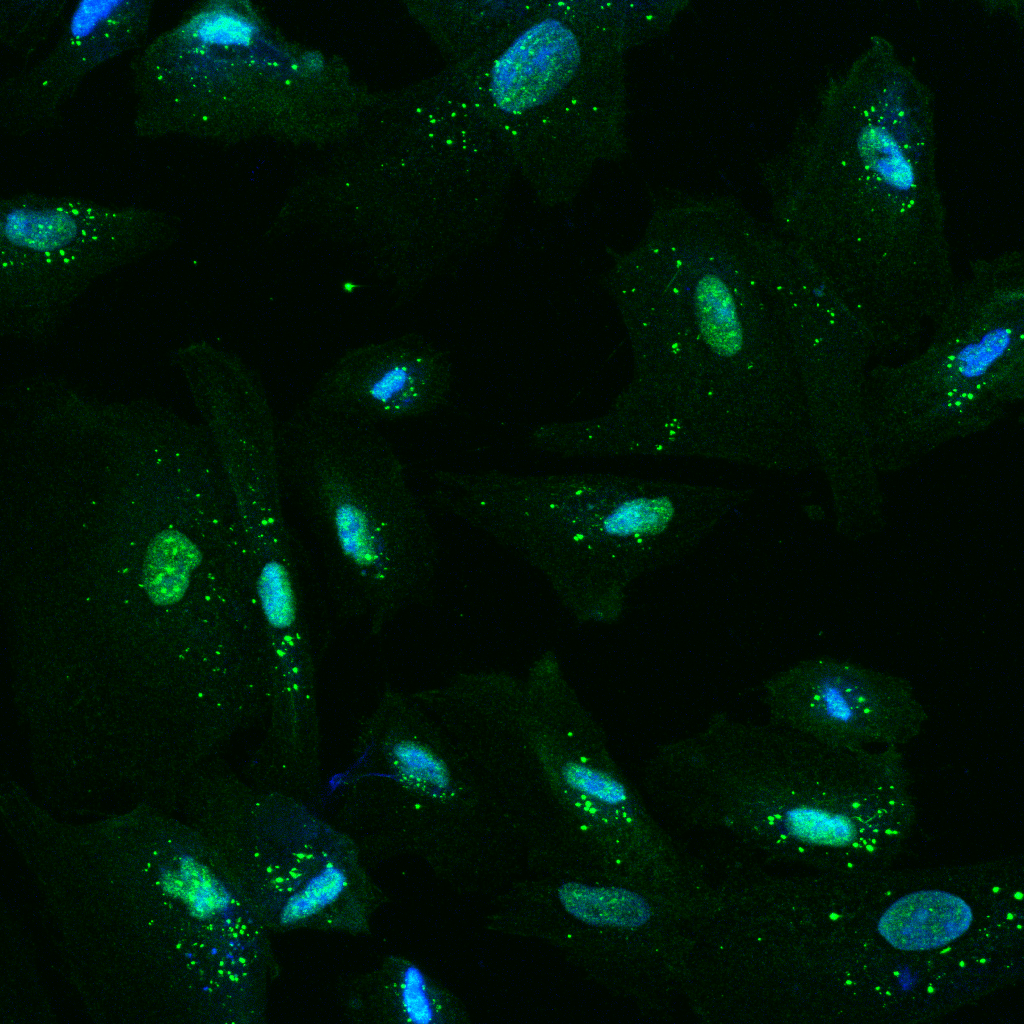

Supplement: S1 File — Characterization of isolated glomerular and tubular ARPCs, showing immunofluorescence of tubular ARPCs with Oct-4 (D), PAX2 (E), BMI-1 (F), and of glomerular ARPCs with Oct-4 (J), PAX2 (K), BMI-1 (L). (ZIP) [file pone.0128258.s001.zip › SC characterization/SC characterization/OCT4/GLOM/Esp28-04-06 Pool_OvPjm1glomeruli Oct4.tif]

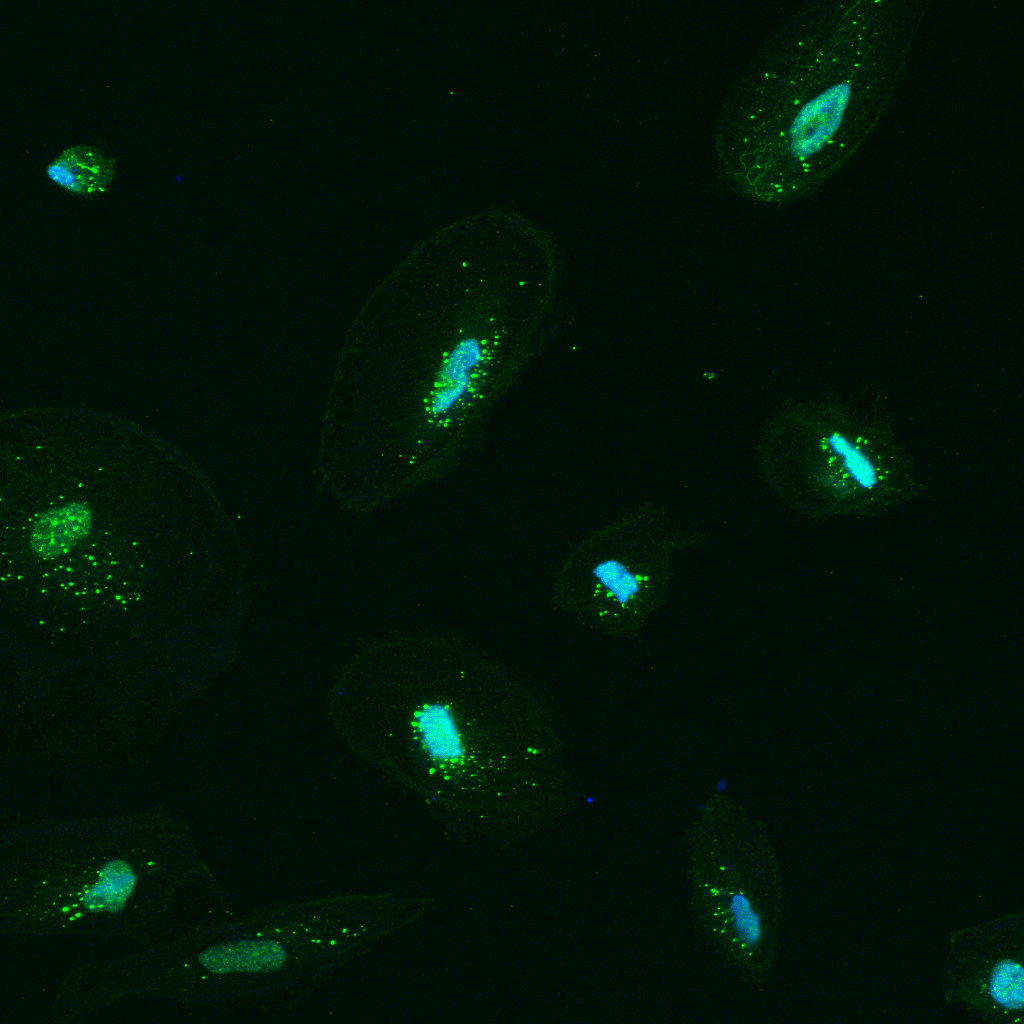

Supplement: S1 File — Characterization of isolated glomerular and tubular ARPCs, showing immunofluorescence of tubular ARPCs with Oct-4 (D), PAX2 (E), BMI-1 (F), and of glomerular ARPCs with Oct-4 (J), PAX2 (K), BMI-1 (L). (ZIP) [file pone.0128258.s001.zip › SC characterization/SC characterization/OCT4/TUB/Esp28-04-06 Pool_OvPjm1 tubuli Oct4.tif]

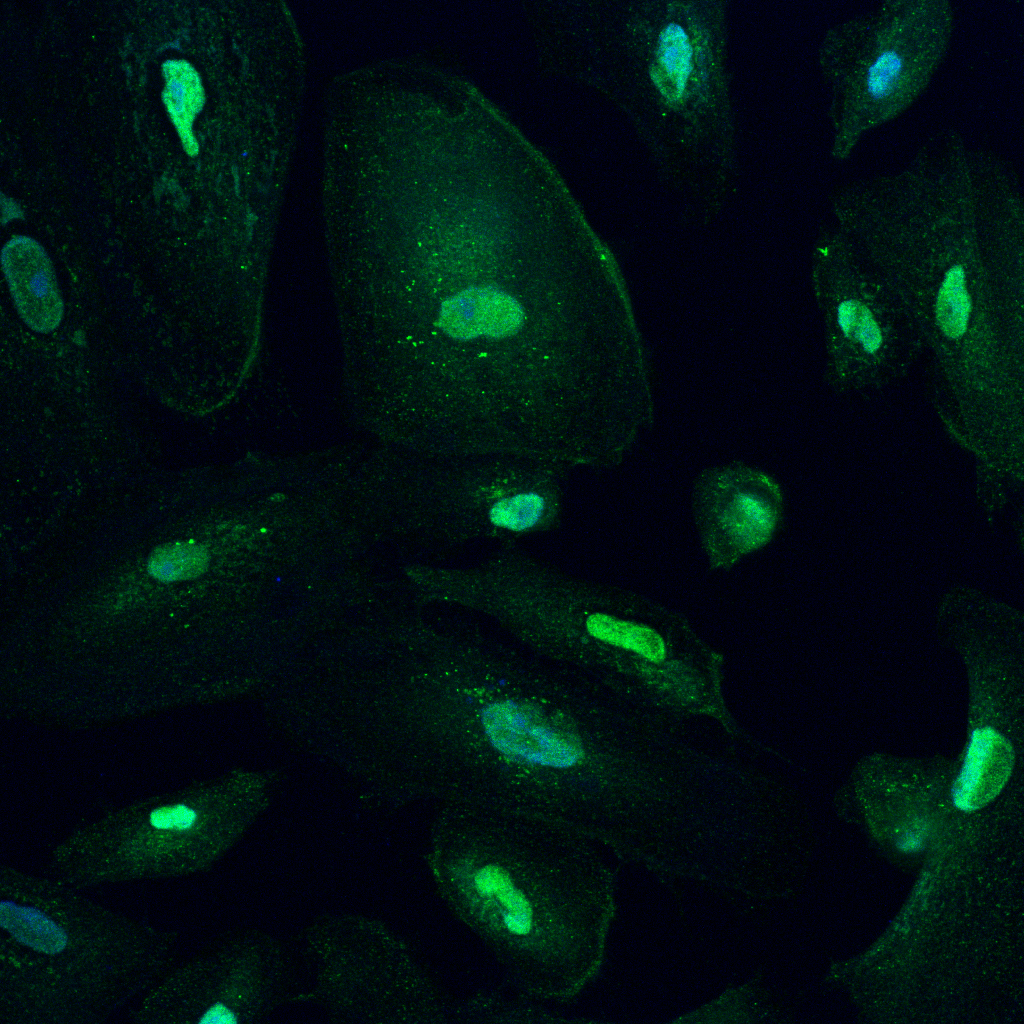

Supplement: S1 File — Characterization of isolated glomerular and tubular ARPCs, showing immunofluorescence of tubular ARPCs with Oct-4 (D), PAX2 (E), BMI-1 (F), and of glomerular ARPCs with Oct-4 (J), PAX2 (K), BMI-1 (L). (ZIP) [file pone.0128258.s001.zip › SC characterization/SC characterization/PAX2/GLOM/Esp28-04-06 Pool_OvPjm1glomeruli PAX2.tif]

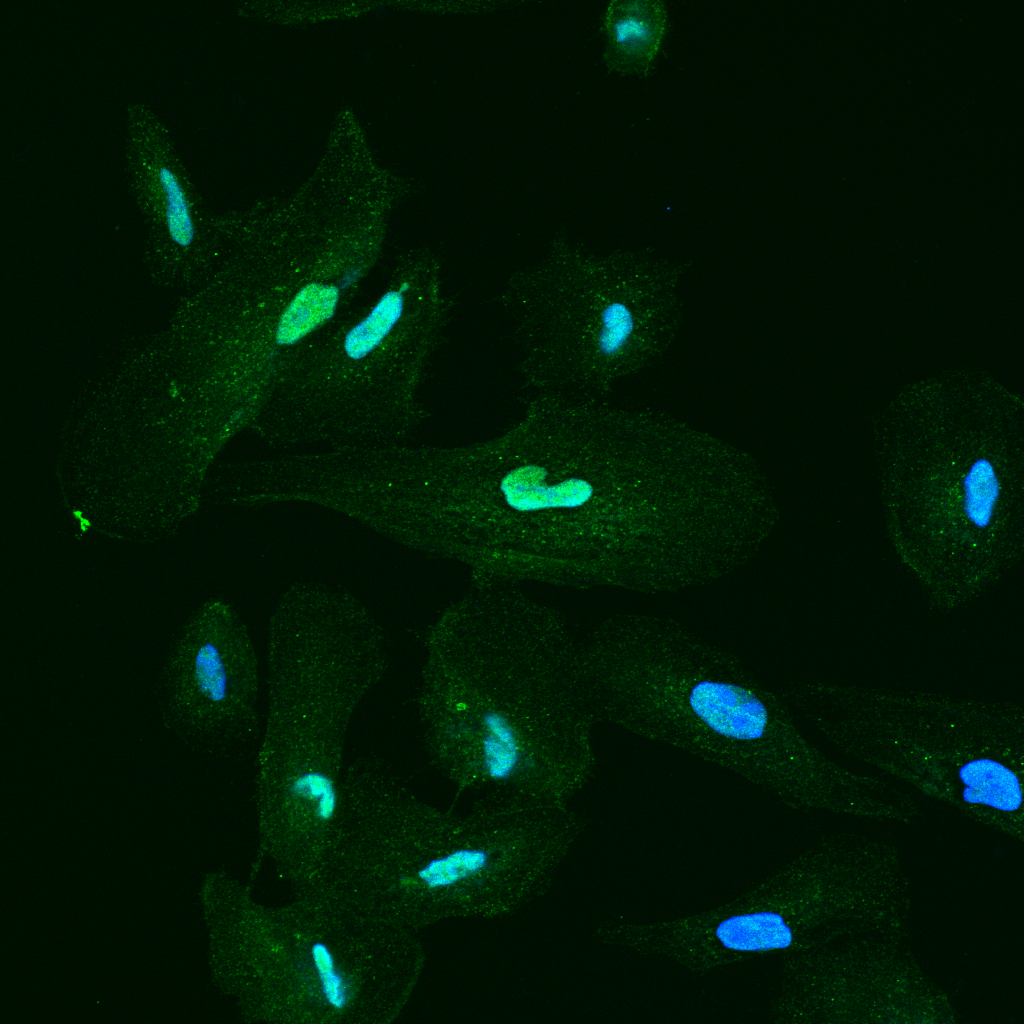

Supplement: S1 File — Characterization of isolated glomerular and tubular ARPCs, showing immunofluorescence of tubular ARPCs with Oct-4 (D), PAX2 (E), BMI-1 (F), and of glomerular ARPCs with Oct-4 (J), PAX2 (K), BMI-1 (L). (ZIP) [file pone.0128258.s001.zip › SC characterization/SC characterization/PAX2/TUB/Esp28-04-06 Pool_OvPjm1 tubuli PAX2.tif]
